# Predicting electronic properties of molecules: a stacking ensemble model for HOMO and LUMO energy estimation

**DOI:** 10.1039/d5ra08007j

**Published:** 2026-03-04

**Authors:** Omid Mahmoudi, Mi-hyun Kim

**Affiliations:** a Department of Pharmacy, Gachon University Incheon South Korea kmh0515@gachon.ac.kr

## Abstract

The energies of the Highest Occupied Molecular Orbital (HOMO) and Lowest Unoccupied Molecular Orbital (LUMO) are important determinants of molecular reactivity and stability. Traditional quantum chemical (QC) methods for calculating the HOMO and LUMO energies face drawbacks, including their costliness and long computational time. Therefore, machine learning is well-positioned to act as a catalyst for QC predictions. In this study, we report a stacking ensemble model named HLP-Stack (HOMO–LUMO predictor *via* stacking) to predict the energy values using molecular descriptors and the QM9 dataset. The stacking achieved robust predictive performance superior to any single model by combining 2D/3D descriptors of the QM9 dataset. It achieved high predictive performance on the test set (*R*^2^ ≈ 9.999 × 10^−1^, RMSE ≈ 3.219 × 10^−4^ Hartree (*E*_h_) for HOMO; *R*^2^ ≈ 9.999 × 10^−1^, RMSE ≈ 1.903 × 10^−4^*E*_h_ for LUMO), outperforming individual baseline models. Feature selection using the SelectKBest algorithm with mutual information regression identified the most influential descriptors. To ensure these descriptors did not trivially encode HOMO or LUMO energies, we performed correlation analysis between each descriptor and the target properties. SHAP Tree Explainer analysis further revealed the feature contribution of each feature to model predictions. In addition, analysis of molecular topology and functional groups highlighted trends in aromaticity and ring structures, and their impact on electronic behavior. Finally, HOMO–LUMO gap analysis demonstrated how molecular structure and functionalization affect electronic properties.

## Abstract 2

A stacking ensemble model for HOMO and LUMO energy estimation.

## Graphical abstract

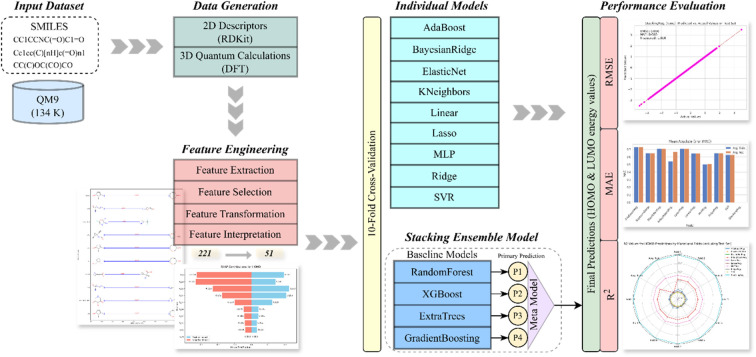

## Introduction

Quantum mechanics provides a fundamental framework for modern chemistry,^1,2^ drug discovery,^3,4^ and machine learning (ML).^1^ It enables accurate computational modelling and elucidation of molecular interactions.^5–7^ Quantum mechanics facilitates the calculation of electronic characteristics,^8^ reaction pathways,^9^ and material properties by explaining the behaviour of matter at the atomic and molecular levels.^8,9^ These calculations are crucial for developing novel molecules and advanced materials.^10,11^ In drug discovery, quantum mechanical computations are significant for accurate prediction of molecular interactions.^4,12,13^ Analysing the electronic properties of molecules facilitates the prediction of their interactions with biological targets. This enables the identification and optimization of drug candidates.^12,14,15^ This process can improve the efficacy and safety of novel pharmaceuticals while streamlining drug development.^3,4,13^ The accurate prediction of highest occupied molecular orbital (HOMO) and lowest unoccupied molecular orbital (LUMO) energies is among the most critical tasks in quantum chemical (QC) calculations.^16,17^

The HOMO–LUMO energy gap correlates with a molecule's reactivity; smaller gaps suggest higher reactivity because less energy is required to excite an electron from the HOMO to the LUMO. Therefore, accurate prediction of the energy gap can facilitate to control broad chemical transformations and biochemical phenomena including materials discovery, photovoltaics, catalyst design, and drug–target interactions.^18–20^ The role of the HOMO–LUMO gap extends beyond organic light-emitting diodes (OLEDs) and organic field-effect transistors (OFETs).^18^ In particular, the gap is a determining factor of optical properties in photovoltaics.^19^ Moreover, it is used as a first-order approximation for the band gap in organic semiconductors.^20^

ML has transformed data-driven approaches, fostering notable advances in pharmaceutics, drug discovery, and molecular science.^3,4,13,21^ The application of ML methods in molecular science has gained substantial momentum, largely due to the demand for efficient and accurate predictive tools. Recent developments highlight the synergy between computational molecular science and ML techniques, particularly in the prediction of atomic properties with quantum-chemical levels of accuracy.^1,22^ ML methods enable property prediction at markedly reduced computational cost compared to that of conventional QC calculations such as Density Functional Theory (DFT).^6^ In comparative assessments, Faber *et al.*^23^ reported that ML models achieved mean absolute errors of approximately 0.02–0.05 eV, notably lower than the 0.1–0.2 eV typically observed for hybrid-DFT predictions relative to experiment, while Fiedler *et al.*^24^ demonstrated that ML surrogates reproduced DFT-level accuracy with errors below 10 meV per atom at orders-of-magnitude lower cost. These findings underscore that ML can achieve DFT-comparable or superior accuracy while drastically reducing computational cost.

This efficiency positions ML models as transformative tools in computational chemistry, underpinning high-throughput discovery and analysis. However, the performance of ML algorithms depends critically on the molecular representations and featurization that encode relevant chemical and physicochemical properties. Effective featurization incorporates diverse information on different chemical structures and their properties, such as bond structures, atomic coordinates, and chemical composition.^25^ ML models utilize these features to map input data to target outputs.^6^ Furthermore, developing domain-specific ML algorithms requires rigorous benchmarking against challengeable datasets.^26^ In cheminformatics, the Simplified Molecular Input Line Entry System (SMILES) is widely adopted as a standard for text-based representation of molecular structures.^27^ SMILES encodes atoms and bonds following explicit syntactic rules, thereby offering computational efficiency while preserving structural validity. It can be directly used for QC calculations of structural motifs and demonstrates the performance of ML models in predicting QC properties. Thus, SMILES-based descriptors suggest an opportunity to integrate QC properties with conventional molecular descriptors. The properties of the compounds in the QM9 dataset were computed utilizing DFT with the B3LYP functional and the 6-31G (2d, f) basis set. It has become a foundational resource for benchmarking ML models for QC calculations.^17,19,28^

In this study, we aimed to clarify the connection between molecular properties of QM9 and SMILES-based descriptors. The combination was aimed at predicting HOMO, LUMO, and the gap between them. These properties pose a challenge for ML algorithms because they rely on complex molecular features, thereby providing a robust test for SMILES-based representations. Consequently, we extracted a comprehensive set of 215 features from the SMILES notation and used them as input for diverse ML algorithms. Furthermore, we employed feature engineering techniques, including correlation-based feature selection, random forest^29^ importance ranking, and mutual information regression, to distinguish key topological descriptors that are most significant to each property. This approach not only enhances predictive performance but also provides insights into the relationship between molecular topology and quantum properties, consequently improving the interpretability of the ML models.^30^

Several prior studies have investigated ML approaches for predicting HOMO–LUMO gap, and related quantum properties using datasets such as QM7b,^17^ QM9, and GDB13.^31^ Reported models differ in dataset size, quantum level of theory, and descriptor representation. Early QSPR-based works^32^ employed small custom datasets with moderate accuracy (*R*^2^ ≈ 0.86), whereas large-scale Hartree–Fock studies^31^ utilized ensemble learning with MAE ≈ 0.166 *E*_h_. Multi-dataset approaches^17^ combining QM7b and QM9 achieved improved efficiency using kernel ridge regression (KRR)^33^ and delta ML^34^ (MAE ≈ 0.1 *E*_h_).

In another study,^35^ SMILES-based feed-forward neural networks (FNN)^36^ were trained to predict multiple quantum-chemical properties from high-dimensional descriptor sets using the open-source Mordred descriptor package^37^ to generate 1826 descriptors and tested five different feature-selection schemes (*e.g.* correlation-based, LASSO,^38^ random forest-based,^29^ and baseline no-selection) to reduce the high-dimensional descriptor set. A multitask ML framework^39^ using fingerprint-based feature vectors (extended-connectivity fingerprint, ECFP)^40^ was also proposed to jointly predict several quantum-chemical targets without manual feature reduction. Table 1 summarizes representative studies focusing on HOMO–LUMO gap prediction across diverse datasets and ML methods.

**Table 1:** Overview of datasets, quantum methods, and machine learning models in related HOMO–LUMO prediction studies

Ref.	Dataset	Quantum method	Features	ML model
32	112 custom organic compounds	DFT (B3LYP/6-31G*)	∼20 atomic “Signature” desc.	QSPR linear regression
31	GDB13 (∼47k)	Hartree–Fock (HF/6-31G(d))	217 RDKit mol. desc.	Ensemble ML
17	QM7b (7k)	ZINDO & GW	∼60 quantum/geometric feat.	KRR & Delta-ML
	QM9 (133k)	DFT (B3LYP/6-31G (2df, p))		
35	QM9 (∼134k)	DFT (B3LYP/6-31G (2df, p))	880 SMILES-based desc.	FNN
39	QM9 (∼134k)	DFT (B3LYP/6-31G (2df, p))	1024-Bit ECFP fp. + GCN embeddings	Stacked single-target & adapted multitask
	Alchemy (12k)			
	Tox21 (8k)			
This work	QM9 (∼134k)	DFT (B3LYP/6-31G (2df, p))	51 selected 2D + 3D mol. desc.	Stacking ensemble ML

Although numerous ML models have been developed for molecular property prediction using the QM9 dataset, most prior studies have relied on single algorithms without model integration or advanced feature optimization. For instance, previous work using FNN-based SMILES-based descriptors^35^ or multitask fingerprint-based frameworks^39^ did not incorporate ensemble learning or systematic feature engineering to reduce dimensionality and enhance efficiency. In contrast, the present study introduces a stacking ensemble model that integrates multiple high-performing regressors (Random Forest,^29^ Extreme Gradient Boosting,^41^ Extra Trees,^42^ and Gradient Boosting^43^) with a linear meta-learner to achieve robust and accurate prediction of HOMO and LUMO energies.

**Fig. 1:**
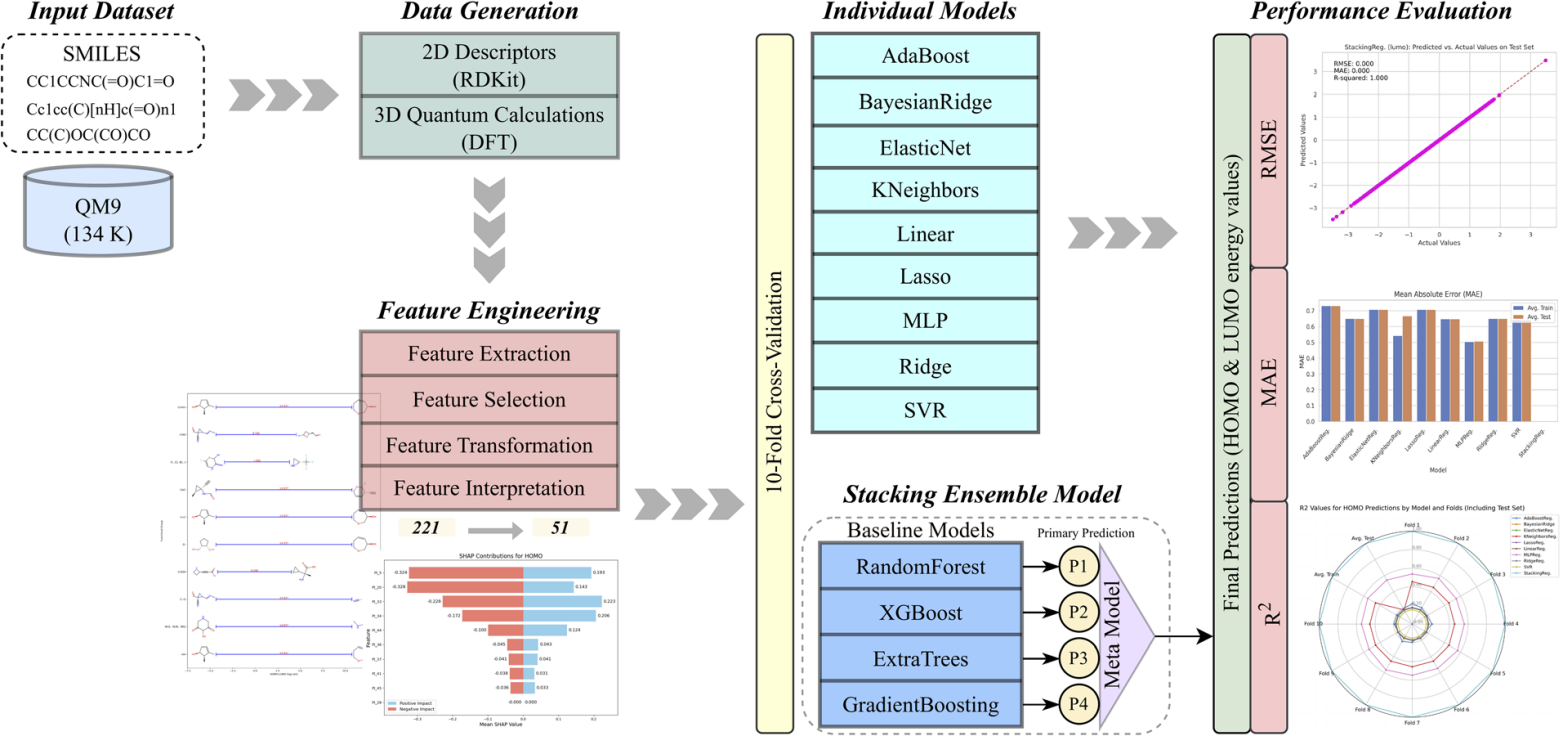
Schematic overview of the model architecture and workflow.

Through a comprehensive feature engineering strategy, we refined the descriptor set to 51 features that capture the most relevant structural and physiochemical information, effectively reducing the feature space while preserving essential molecular properties. To ensure the selected descriptors did not trivially encode HOMO or LUMO energies, we performed correlation analysis between each descriptor and the target properties, confirming the absence of data leakage or redundant dependencies. The reduced feature set offers several advantages, including faster computation, lower memory usage, reduced processing cost, and minimize overfitting, while maintaining high predictive accuracy. To the best of our knowledge, no stacking ensemble model has been developed for predicting HOMO and LUMO energies on the QM9 dataset, making this work a distinctive and efficient contribution to quantum-chemical property prediction. Fig. 1 presents a detailed schematic of the models used in this study, outlining the overall workflow and the interactions among different components.

## Data and methods

### Preparation of molecular dataset

The QM9 dataset, presented by Ramakrishnan *et al.* in 2014, is a broadly utilized benchmark within the ML community.^17,19,28^ It contains the data of over 130 000 organic molecules, each composed of up to nine heavy atoms (C, N, O, and F). This dataset includes the respective geometries, as well as thermodynamic and electronic properties of each molecule. While preprocessing the SMILES data from the QM9 dataset, a few steps were implemented to ensure the integrity and consistency of the molecular representations. The primary step included neutralization of charges on molecules to standardize their electronic states over the dataset. Tautomeric shapes were canonicalized to remove all redundancy and variability, thereby preparing a reliable representation of each molecule. Hydrogens were eliminated to simplify the molecular representations, consequently improving computational efficiency while maintaining the essential structure of the molecules. Additionally, isotopic labels were cleared to focus on the core molecular graphs. Stereochemistry was either preserved or standardized based on the preprocessing requirements. Finally, a sanitization step guaranteed aromaticity and valence states for each molecule, and other chemical properties were preserved. Following these preprocessing steps, each molecule was converted to a canonicalized SMILES representation.

### Data generation and feature extraction

A comprehensive set of molecular descriptors was generated to capture the structural, electronic, and physicochemical descriptors of the molecules. Two-dimensional (2D) molecular properties were calculated utilizing the RDKit library,^27,44^ resulting in 208 different descriptors derived from SMILES representations of the molecules, including Morgan fingerprints,^27,40^ electro-topological state, ring structures, van der Waals surface areas, functional group counts, hydrophobicity and polarity metrics, and steric and structural properties. Additionally, three-dimensional (3D) molecular descriptors were chosen from the QM9 (ref. 28) dataset, calculated from at the B3LYP/6-31G (2df, p) level of theory. By combining the 2D and 3D descriptors, the initial feature set, comprising 221 distinct molecular descriptors along with target values from QM9, was established.

### Feature selection

For reducing feature dimensionality, features were correlated with the target variables to prevent overfitting and enhance computational efficiency.^9,45^ In the step, the informative features were detected for the prediction of HOMO and LUMO energies. At first, a wide set of features was selected based on their theoretical relevance to the molecular energies. To further improve the performance of the model and dimensional reduction, a systematic approach was utilized *via* the SelectKBest^46^ algorithm; mutual information regression^30^ was used as the scoring function. Mutual information computes the statistical relationship between features and target variables without considering any specific distribution, making it robust for nonlinear dependencies. Because mutual information is non-parametric, the scaling of features does not directly affect the feature selection process.^24^ The *K* number of 100 deemed most important and relevant to the prediction of HOMO energy were selected. These descriptors were reviewed to ensure they represent key molecular features that contribute to the target property. A similar approach was applied for LUMO energy, resulting in another set of 100 top descriptors. The descriptor sets for HOMO and LUMO were combined to identify common important features. A combination of the two sets yielded 59 consistently important features over both targets. These common descriptors were considered the foremost important features for building predictive ML models. This determination not only decreased the dimensionality of the dataset but also ensured that the selected features provided a balanced representation for HOMO and LUMO predictions.

### Feature transformation

After compilation of the feature set, feature transformation techniques were applied to ensure the data was suitable, improved the comparability of features, and facilitated efficient ML model training. This step included two important operations—standardization and normalization—that were applied to the selected features and target variables. Normalization was applied to adjust some features to a range of [0, 1], which is useful for algorithms sensitive to feature scale. In addition, the features were standardized to have a mean of 0 and a standard deviation of 1 to ensure that they were on the same scale to decrease the bias towards features with larger scales and indicated better model performance during the regression tasks. Features with zero variance were removed due to the absence of meaningful information and noise issue. After elimination, the final set of features comprised 51 refined features and two target properties, HOMO and LUMO energies, prepared for ML model development. The selected descriptors provide insight into electronic and structural factors affecting molecular properties and reactivity, as summarized in the SI (Table S1).

To further ensure that the selected molecular descriptors did not trivially encode or directly correlate with the target properties, we evaluated the Pearson and Spearman correlations between each descriptor and HOMO and LUMO energies. No descriptor exhibited a correlation coefficient above |*r*| = 0.95, confirming the absence of trivial dependencies or data leakage. The strongest Pearson correlation observed for HOMO was with the molecular polarizability (alpha, *r* = 0.24), following by the zero-point vibrational energy (zpve, *r* = 0.19), whereas for LUMO the highest correlation was with zpve (*r* = 0.64) and dipole moment (mu, *r* = 0.40). These moderate correlations are physically meaningful, as they reflect the shared influence of molecular size, geometry, and charge distribution on electronic properties, yet they are far from direct or one-to-one relationships.

**Fig. 2:**
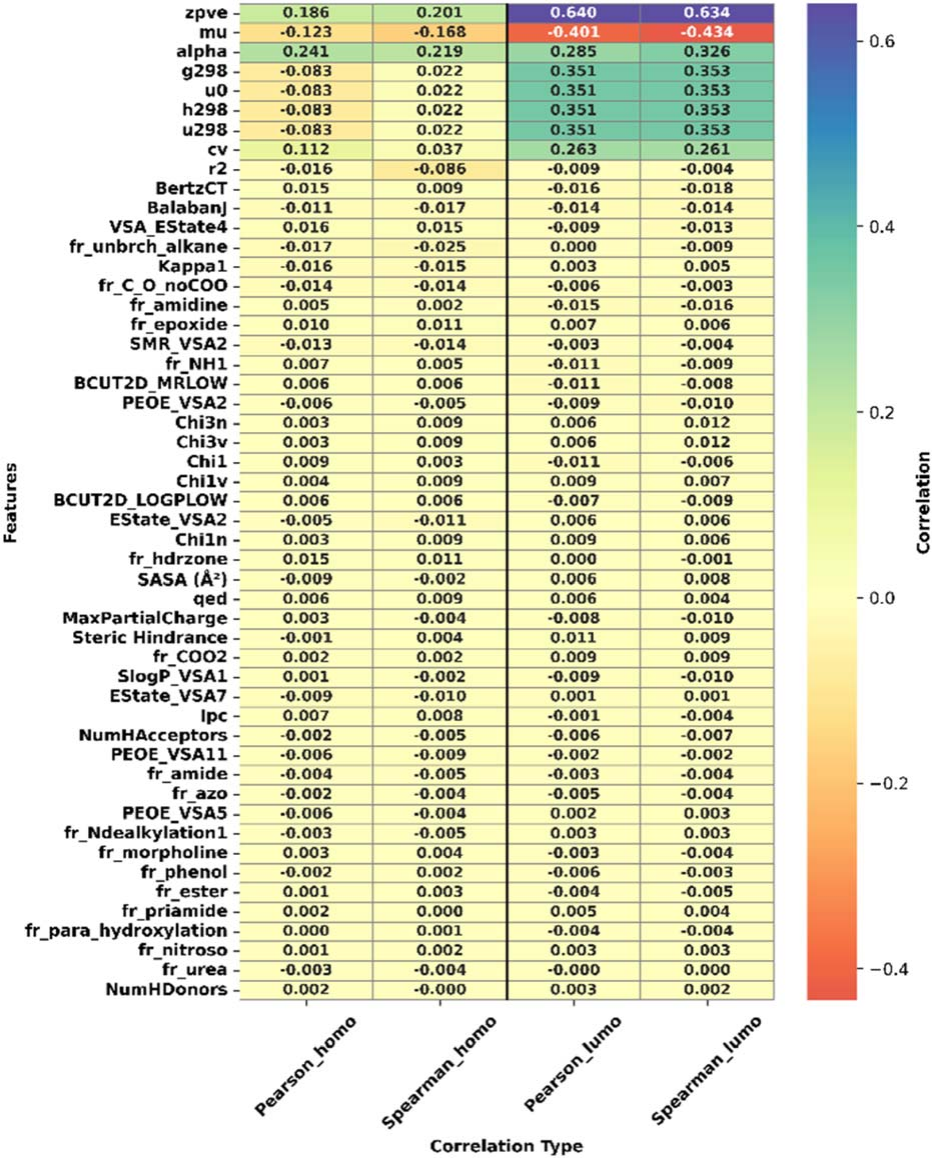
Heatmap of feature correlations with HOMO and LUMO energies. Pearson and Spearman correlation coefficients are shown for each descriptor. Red indicates positive correlation, and blue indicates negative correlation. The absence of any |*r*| ≥ 0.95 confirms that the selected descriptors are not trivially related to the target properties.

The heatmap in Fig. 2 provides a visual summary of these correlations. The heatmap rows (features) are sorted by their average absolute correlation across all four correlation types, meaning that features most strongly correlated (positively or negatively) with either HOMO or LUMO appear at the top, facilitating visual interpretation of their relative importance. Overall correlation analysis indicates that the selected descriptors capture complementary structural, topological, and electronic information without overlapping with the quantum-mechanical target values. Consequently, the model learns non-trivial and chemically relevant relationships between molecular features and orbital energies rather than relying on redundant or directly encoded information. This validates that the descriptor set is appropriate for predicting HOMO and LUMO energies while maintaining physical interpretability and statistical independence from the targets.

Furthermore, we performed a comprehensive correlation analysis among these features to evaluate their independence. The correlation matrix was computed and visualized in Fig. S1A, while the corresponding heatmap highlighting strong correlations (|*r*| > 0.8) is presented in Fig. S1B. As illustrated, only a limited number of descriptors exhibited moderate to strong correlations (mainly within related descriptor families such as topological indices and energy-based variables). The majority of features demonstrated low pairwise correlations, indicating that most descriptors were statistically independent and suitable for reliable machine learning model construction without multicollinearity issues.

To further confirm this, Random Forest Regressor^29^ models were trained for HOMO and LUMO energy prediction using both the complete feature set (51 descriptors) and the correlation-reduced dataset. The nearly identical RMSE values for HOMO (0.000132 *vs.* 0.000137) and LUMO (0.000076 for both) demonstrate minimal redundancy among descriptors and confirm that the selected features provide robust, non-overlapping information for highly accurate HOMO and LUMO energy prediction.

**Fig. 3:**
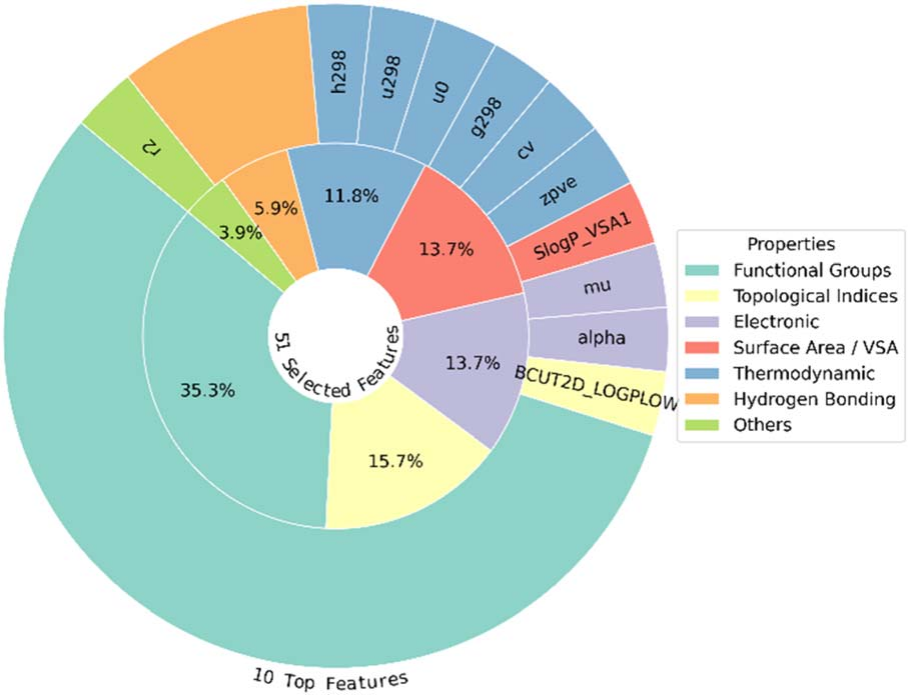
Descriptor composition by functional category: the inner pie shows all selected features, whereas the outer pie shows the top 10 features.

To elucidate the role of descriptors, they were grouped into functional categories based on their chemical or physical roles. Fig. 3 presents this classification and indicates the composition and distribution of descriptor types used in the analysis. This simplified method preserved interpretability and computational efficiency. Simultaneously, it ensured that the dataset was optimized for predictive performance by achieving a balance between the preservation of important information and reduction of dimensionality. The inner pie chart represents the distribution of all selected features across functional categories, whereas the outer pie chart highlights the top 10 most important features.

### Feature interpretation

The SHapley Additive exPlanations (SHAP)^47^ Tree Explainer is used to interpret feature importance. Each target variable has its explanation, which calculates SHAP values that indicate the contribution of each feature to the corresponding predictions. These values provide a clear breakdown of the model's output, thereby enabling a comprehensive analysis of the predictive significance of each molecular descriptor.

**Fig. 4:**
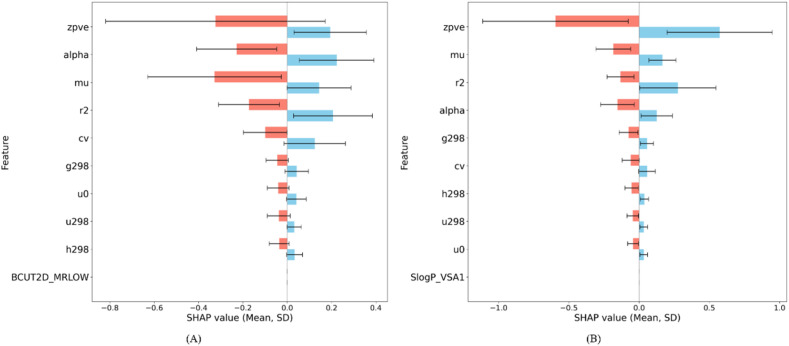
Top 10 features based on SHAP values for (A) HOMO and (B) LUMO energy (positive SHAP: blue, negative SHAP: red).

The value prediction of the SHAP summary plots for HOMO and LUMO are shown in Fig. 4, ranking the top 10 features based on their mean absolute SHAP value and standard deviation. These plots highlight the most crucial descriptors; higher SHAP values indicate a stronger contribution to the model's prediction. Positive SHAP (blue) values suggest an increase in the target variable, whereas negative values (red) indicate a decrease. Comparison of the two plots revealed features that significantly affect both HOMO and LUMO energy values, as well as those that primarily affect only one target.

SHAP analysis revealed that the zero-point vibrational energy was the most influential descriptor for both HOMO and LUMO predictions. Moreover, key descriptors such as polarizability (alpha) and dipole moment (mu) also demonstrated significant but directionally varied effects on the predicted values. For HOMO, features such as mu, r2, and cv showed predominantly negative SHAP values, indicating a general lowering effect on HOMO energy. In contrast, the LUMO model exhibited more balanced or slightly positive contributions from the same features. These findings emphasize the distinct feature priorities for HOMO and LUMO energy predictions.

Here, we further explored the mechanisms through which molecular topology, including the presence of polycyclic aromatic hydrocarbons (PAHs), non-aromatic ring systems, and key functional groups, contributes to the structural and chemical diversity of the dataset. We identified trends in aromaticity and ring count by classifying molecules based on their SMILES representations. Similarly, a parallel analysis of functional groups provided additional insight into their potential reactivity and physicochemical properties.

We classified molecules based on their canonical SMILES representations to categorize the molecular structures in our dataset, focusing on the presence of PAHs and non-PAH ring structures. The classification criteria were as follows: molecules containing a single benzene ring, naphthalene-like molecules with two aromatic rings, anthracene-like molecules with three aromatic rings, large PAHs with four or more aromatic rings, and molecules with non-aromatic ring systems or no rings at all.

**Fig. 5:**
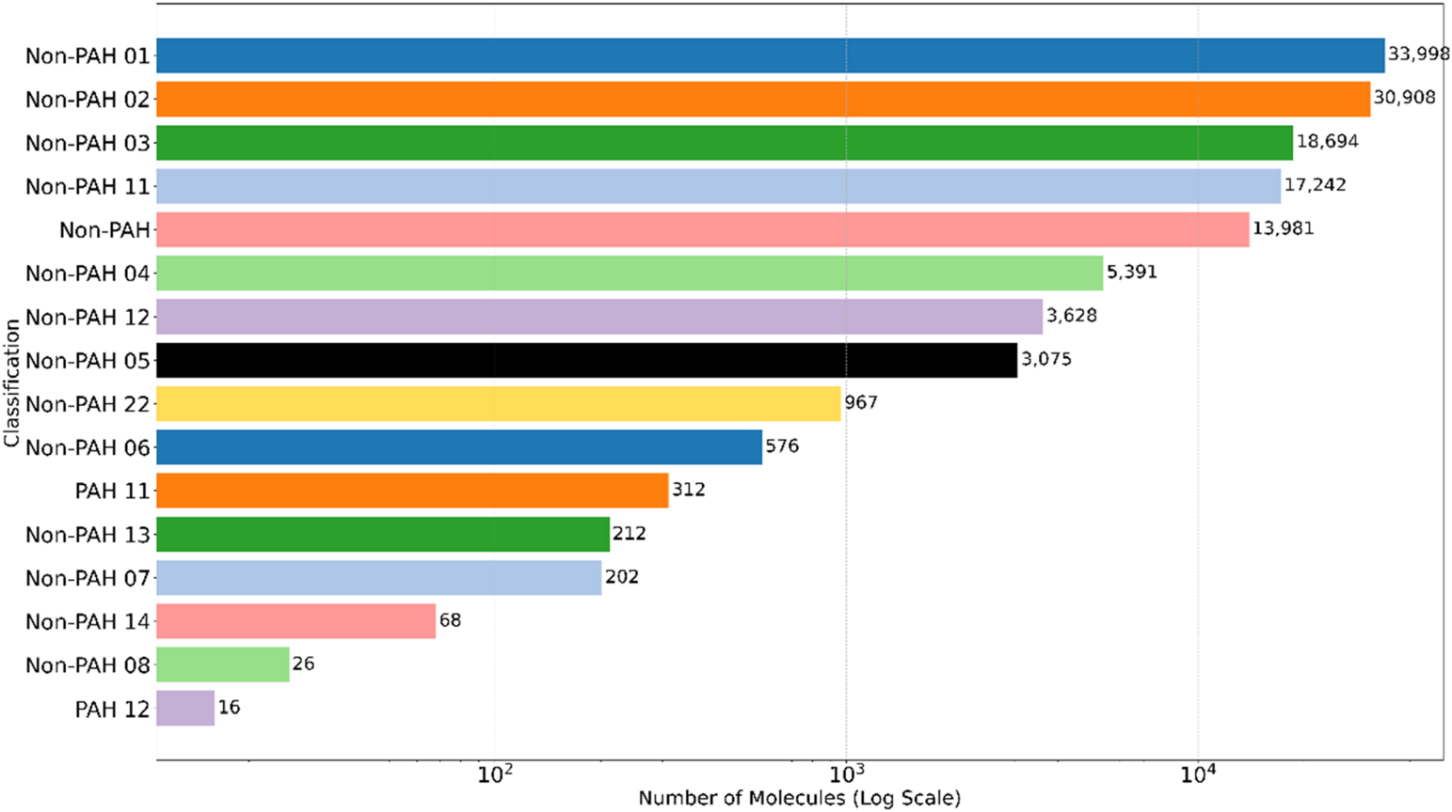
Classification of molecules based on aromaticity and ring structures. For PAH labels, the first and second numbers denote benzene rings and aromatic rings (*e.g.*, PAH 11 = 1 benzene + 1 aromatic ring), respectively. For non-PAH labels, the first and second numbers denote aromatic and total rings (*e.g.*, non-PAH 23 = 2 aromatic rings within 3 total rings), respectively.

Non-PAH structures dominate the dataset, with the largest subgroup consisting of molecules with one non-aromatic ring (33 998 molecules). In contrast, aromatic molecules are comparatively rare, with only 312 classifieds as PAH-1 (single benzene, one aromatic ring). The dataset contains considerably few large PAHs, as no molecules with more aromatic rings were detected under the current classification. However, multi-ring systems are prevalent in the non-PAH category; some molecules contained up to eight non-aromatic rings. A detailed breakdown of these classifications is shown in Fig. 5, providing further insight into the structural diversity of the data. We subsequently created multiple datasets by combining the classifications from aromaticity and ring structures with the presence of specific functional groups. For instance, a dataset consists of molecules classified as non-PAH (no rings) that contain the –OH (alcohol) functional group. This process was repeated for all combinations of the classified molecules (Fig. 5) and various functional groups, resulting in a comprehensive analysis of structural diversity.

The HOMO–LUMO gap of the molecules in each dataset was plotted as a function of the number of carbon atoms in the molecule to further explore electronic properties. This visualization provides insights into the effects of both the molecular framework (aromaticity and ring structures) and functionalization (functional groups) on electronic behaviour.

**Fig. 6:**
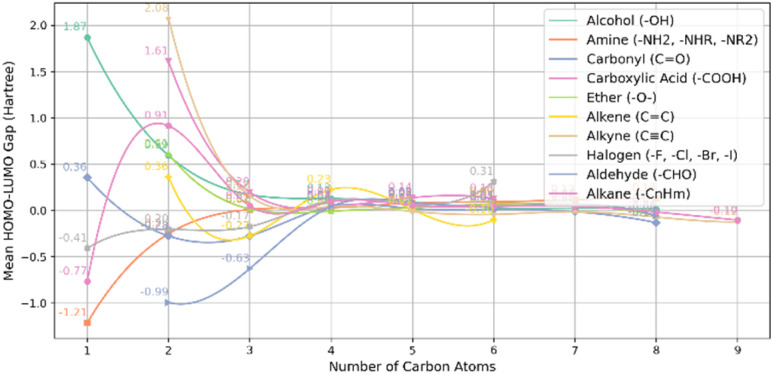
Mean HOMO–LUMO gap *vs.* carbon count for non-PAH (no rings) and various functional groups.

The comparison plot shown in Fig. 6 demonstrates the HOMO–LUMO gap for molecules classified as non-PAH (no rings), each containing different functional groups. The maximum HOMO–LUMO gap is found in alkyne (C≡C) groups, with a value of 2.08 *E*_h_ for molecules containing two carbon atoms. This is followed closely by the alcohol (–OH) group, which has a gap of 1.87 *E*_h_ with only one carbon atom. In contrast, the amine (–NH_2_, –NHR, and –NR_2_) group exhibits the minimum HOMO–LUMO gap of −1.21 *E*_h_ for molecules with only one carbon atom. As the number of carbon atoms increases, the HOMO–LUMO gap gradually decreases for all functional groups, eventually converging to a value close to zero. For example, the gap reaches −0.10 *E*_h_ for both alkyne (C≡C) and alkane (–C_*n*_H_*m*_) groups when there are nine carbon atoms in the molecule.

**Fig. 7:**
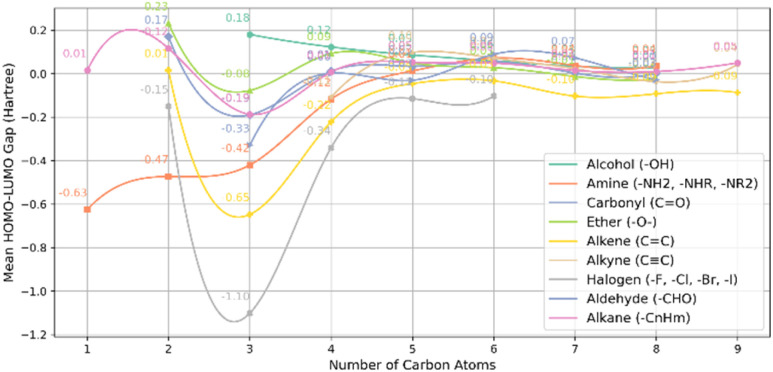
Mean HOMO–LUMO gap *vs.* carbon count for non-PAH (zero aromatic rings, one total rings) and various functional groups.

These results indicate that small molecules with fewer carbon atoms tend to have larger HOMO–LUMO gaps, meaning they are less electronically stable and more reactive. For example, the alkyne (C≡C) and alcohol (–OH) groups show higher gaps with fewer carbon atoms. As the molecules get larger, their electronic structure becomes more stable, and the HOMO–LUMO gap shrinks. The amine (–NH_2_, –NHR, and –NR_2_) group has a negative gap for smaller molecules. This suggests an electron-donating effect, which lowers the energy difference between the HOMO and LUMO. Ultimately, the gap approaches zero for larger molecules, indicating electronic stabilization and reduced reactivity, especially for alkyne and alkane groups with nine carbon atoms. Fig. 7 shows a comparison between non-PAH (zero aromatic rings, one ring) and various functional groups. For molecules with no aromatic rings and only one ring, the smallest HOMO–LUMO gap is −1.10 *E*_h_, observed in the halogens (–F, –Cl, –Br, –I) with three carbon atoms. In contrast, the largest gap of 0.22 *E*_h_ is seen in the ethers (–O–) with two carbon atom.

Similar to non-PAH (no rings) molecules, the HOMO–LUMO gap tends to approach zero as the number of carbon atoms increases in these compounds. For instance, molecules with alkyne (C≡C) and alkane (–C_*n*_H_*m*_) functional groups exhibit a gap that diminishes with increasing carbon atoms, reaching a value close to zero (0.05 *E*_h_). The relationship between the mean HOMO–LUMO gap and the number of carbon atoms for other molecules featuring diverse ring systems and functional groups are illustrated in the SI (Fig. S4–S15).

**Fig. 8:**
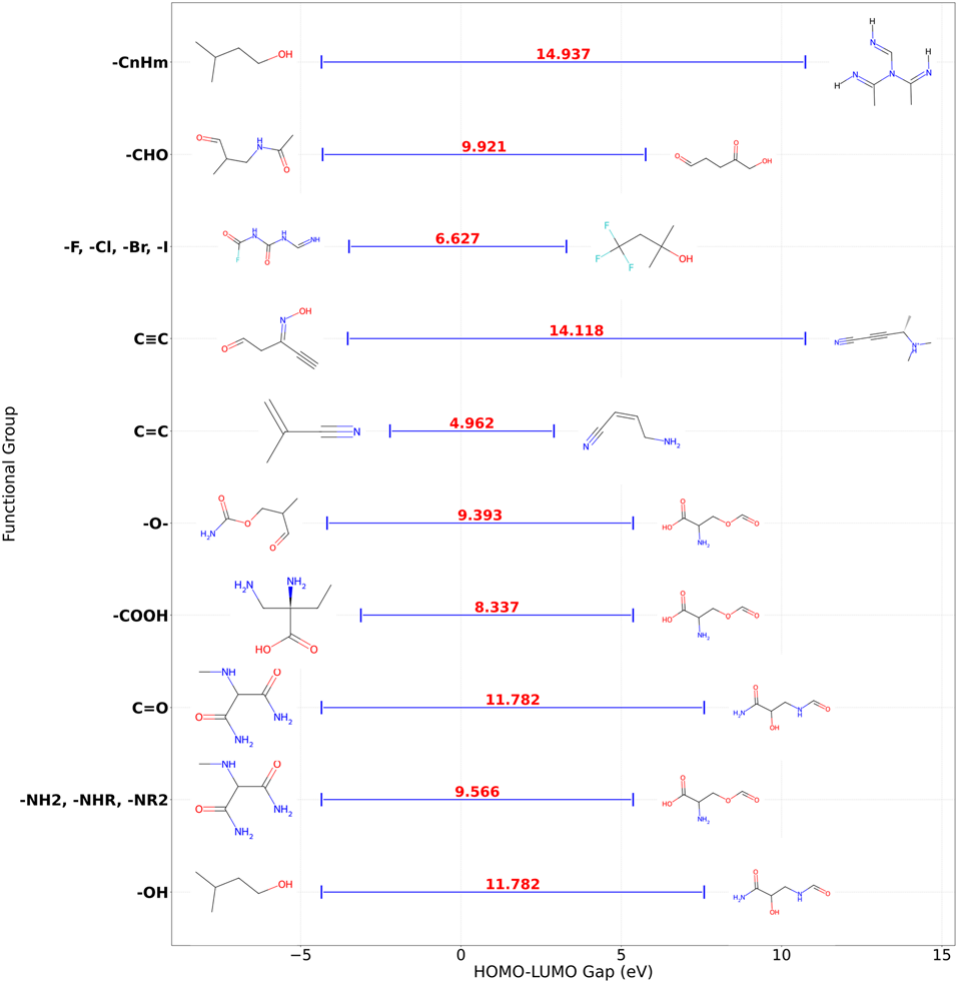
Visualization of HOMO–LUMO gap with annotated molecular structures for non-PAH (no rings).

We further visualized the HOMO–LUMO gaps using a plot that adjusted the vertical spacing between the functional groups based on the number of entries per group. The molecules with the minimum and maximum HOMO–LUMO gaps were identified for each functional group, and 2D structures of these molecules were generated and annotated on the plot. The 2D structures, based on the canonical SMILES of the molecules, provided a visual representation of the structural diversity of the molecules within each functional group. Fig. 8 represents the relationship gap for molecules classified as non-PAH (no rings). The plotted data points correspond to the minimum and maximum HOMO–LUMO gap of two molecules for each functional group. Additionally, the line connecting the two values in the figure indicates the difference between them; it is a visual representation of the gap variation within each functional group. A visualization of the HOMO–LUMO energy gap across the different functional groups and molecular frameworks in presented in the SI (Fig. S16–S30).

### ML algorithms

Selecting the appropriate algorithm is crucial for achieving high prediction accuracy in ML. In this study, multiple regression models were employed to predict the target variables, incorporating a diverse set of approaches, including linear, ensemble, and neural network-based regressors. Nine ML-based regression models were employed for target prediction, optimization of loss functions, and effective generalization. These models include Adaptive Boosting Regressor (AdaBoostRegressor),^48^ Bayesian Ridge Regression (BRR),^49^ Elastic Net Regression (ENet),^50^*K*-Nearest Neighbours Regressor,^51^ Linear Regression (LR),^52^ Least Absolute Shrinkage and Selection Operator (Lasso) Regression,^38^ Multi-Layer Perceptron Regressor (MLPR),^53^ Ridge Regression (RR),^54^ and Support Vector Regression (SVR).^55^ Each model was selected for its unique ability to handle diverse data characteristics and its potential to provide complementary predictions. Meanwhile, a stacked ensemble learning algorithm integrating multiple base learners into a meta-learning framework was implemented to simultaneously enhance predictive accuracy for HOMO and LUMO energy values. The stacking method leverages the strengths of individual regressors by integrating their predictions, often resulting in superior overall performance.^56^

Unlike conventional ensemble methods such as bagging or boosting, which aggregate or sequentially refine model outputs, stacking employs a meta-learner that learns how to optimally combine the predictions from multiple base models, enabling it to capture complementary information among learners.^43,57–59^

This ensemble strategy captures complex relationships in the data more effectively, ensuring robust and accurate predictions for target properties.^56^ To streamline model selection and identify high-performing algorithms, the Lazy Regressor library was employed on the training and validation data.^60^ The stacking regressor (HLP-Stack) in this study incorporated four base models based on the high-performing algorithms identified by Lazy Regressor: Random Forest,^29^ Extreme Gradient Boosting (XGBoost),^61^ Extra Trees,^62^ and Gradient Boosting.^43^ These models were selected for their strong predictive capabilities and complementary strengths in handling complex, high-dimensional data. The outputs from these base models were then used as features for a final meta-estimator—Linear Regression^52^—which combined the learned patterns from base regressors to enhance overall prediction accuracy. The theoretical background of the MLs employed in this study is further described in the SI (see section Machine Learning Models). While the Lazy Regressor framework was efficient for model selection, leading to a robust stacking ensemble, the stacking approach improved generalization, reduced bias and variance, and effectively captured complex patterns in the data. As a result, it achieved high accuracy in predicting HOMO and LUMO energies, thereby ensuring reliable and comprehensive estimation of electronic properties. After applying feature engineering techniques to the QM9 dataset, we constructed a benchmark dataset consisting of 51 molecular descriptors for each compound. The refined dataset was carefully divided into training, validation, and test subsets to ensure a robust evaluation of the predictive models. Initially, 80% of the data, corresponding to 103 436 molecules, were allocated for training, whereas the remaining 20% (25 860 molecules) were reserved as a holdout set. This holdout set was then equally split into 12 930 molecules for validation and testing to facilitate model tuning and final evaluation (Table 2).

**Table 2:** Benchmark datasets demonstration

Dataset	Training	Validation	Test
QM9	103 436	12 930	12 930

Various regression models were implemented using this reduced feature set to predict the HOMO and LUMO energies.^29,38,41–43,46,50^ We employed 10-fold cross-validation^63,64^ to further enhance the reliability of the models. This process involved randomly shuffling the dataset and dividing it into 10 equal parts (or folds). In each iteration, nine folds were used for training and one for testing. This ensured that every data point was utilized for both training and testing exactly once, offering a comprehensive assessment of the models' performance.^65,66^ During this procedure, each regression model had multiple training and testing cycles across all 10 folds. Performance metrics, including *R*^2^, RMSE, and MAE, were recorded at each iteration and averaged to provide an overall evaluation of model performance. This method effectively reduced potential bias or overfitting that may arise from relying on a single dataset split. Thus, it provided a more accurate and generalized view of the models' predictive capabilities. Fig. 1 illustrates the complete architecture and structure of the models employed in this study, providing a schematic overview of the workflow and interactions between the various components.

## Results and discussion

### Workflow overview and evaluation metrics

The workflow of this data-driven analysis encompasses several key stages, including data generation, feature engineering, feature interpretation, ML model selection, and stacking ensemble learning. The dataset was randomly partitioned into training, validation, and test sets, with 80%, 10%, and 10% of the data allocated to each subset, respectively. To assess model performance, multiple evaluation metrics were employed, including *R*^2^, RMSE, and MAE. These metrics collectively facilitate the evaluation of prediction accuracy and quantification of prediction errors.

### Performance of HOMO and LUMO energy predictions

A summary of the performance results for nine state-of-the-art ML models, alongside the ensemble model, in predicting HOMO energy on the test set is presented in Table 3. The ensemble model delivered the best performance, achieving perfect accuracy in predicting HOMO energy. In comparison, the ENet and Lasso models showed the lowest predictive performance among the evaluated models. The performance of LUMO predictions on the test set is presented in Table 4, which includes nine different models alongside the ensemble model. The HLP-Stack model demonstrated superior performance, achieving an RMSE and MAE of 0 and an *R*^2^ of 1. This result significantly outperformed individual models, such as MLP, LR, and BRR, highlighting the effectiveness of the ensemble approach.

**Table 3:** Performance comparison of HOMO predictions on the test set

Model	RMSE (*E*_h_)	MAE (*E*_h_)	*R* ^2^
**HLP-Stack**	**3.219 × 10** ^**−4**^	**6.436 × 10** ^**−5**^	**9.999 × 10** ^**−1**^
MLPReg.	0.685	0.515	0.520
AdaBoostReg.	0.891	0.724	0.188
LinearReg.	0.899	0.646	0.174
BayesianRidge	0.904	0.650	0.165
RidgeReg.	0.904	0.650	0.165
KNeighborsReg.	0.910	0.671	0.154
SVR	0.914	0.641	0.147
ElasticNetReg.	0.989	0.703	0
LassoReg.	0.989	0.703	0

**Table 4:** Performance comparison of LUMO predictions on the test set

Model	RMSE (*E*_h_)	MAE (*E*_h_)	*R* ^2^
**HLP-Stack**	**1.903 × 10** ^**−4**^	**4.740 × 10** ^**−5**^	**9.999 × 10** ^**−1**^
MLPReg.	0.539	0.426	0.711
LinearReg.	0.589	0.470	0.654
BayesianRidge	0.636	0.513	0.597
RidgeReg.	0.636	0.513	0.597
SVR	0.641	0.507	0.590
AdaBoostReg.	0.653	0.565	0.575
KNeighborsReg.	0.767	0.607	0.414
ElasticNetReg.	0.945	0.778	0.110
LassoReg.	1.002	0.823	0

### Performance visualization across datasets

**Fig. 9:**
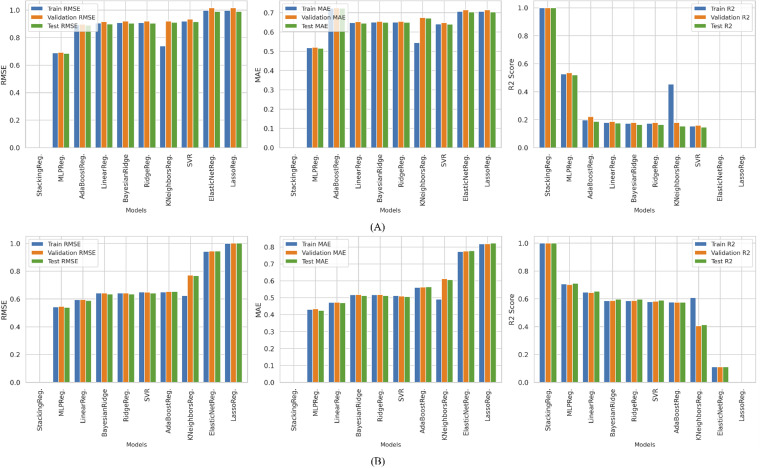
Performance comparison of (A) HOMO and (B) LUMO predictions on all datasets.

Fig. 9 illustrates the performance of various models based on RMSE, MAE, and *R*^2^ values across the training, validation, and test datasets for predicting HOMO and LUMO energy. The HLP-Stack model outperformed all other models, achieving RMSE and MAE values of zero across all datasets. This indicated perfect predictive accuracy. Among individual models, the MLP Regressor demonstrated a relatively strong performance on the test dataset. RMSE, MAE, and *R*^2^ values of 0.536 *E*_h_, 0.515 *E*_h_, and 0.250, respectively, were recorded for HOMO energy, whereas 0.539 *E*_h_, 0.460 *E*_h_, and 0.711, respectively, were recorded for LUMO energy. These results suggest that although MLP captured some patterns within the data, it exhibited a moderate level of error and did not generalize as well as the ensemble model.

For HOMO energy, AdaBoost,^48^ LR,^52^ and BRR^49^ followed MLP in predictive performance across all datasets, indicating their effectiveness in capturing the underlying trends. However, AdaBoost ranked seventh among the ten evaluated models for LUMO energy. This suggests that although it performed well for HOMO energy, its ability to generalize to LUMO energy was comparatively weaker. This difference can be attributed to variations in the dominant SHAP features: while HOMO-related descriptors such as zpve, alpha, mu, r2, and g298 exhibit smoother, quasi-linear dependencies with the target, LUMO-associated features such as Slog *P*_VSA1, mu, and alpha introduce stronger nonlinear and independent interactions related to molecular polarity and charge distribution. Because AdaBoost relies on shallow decision trees, simple base learners with limited depth that capture only low-order feature-response mappings, its ability to model these complex nonlinear interactions is reduced, leading to weaker generalization for LUMO energy.

### 10-Fold cross-validation analysis of model performance

**Fig. 10:**
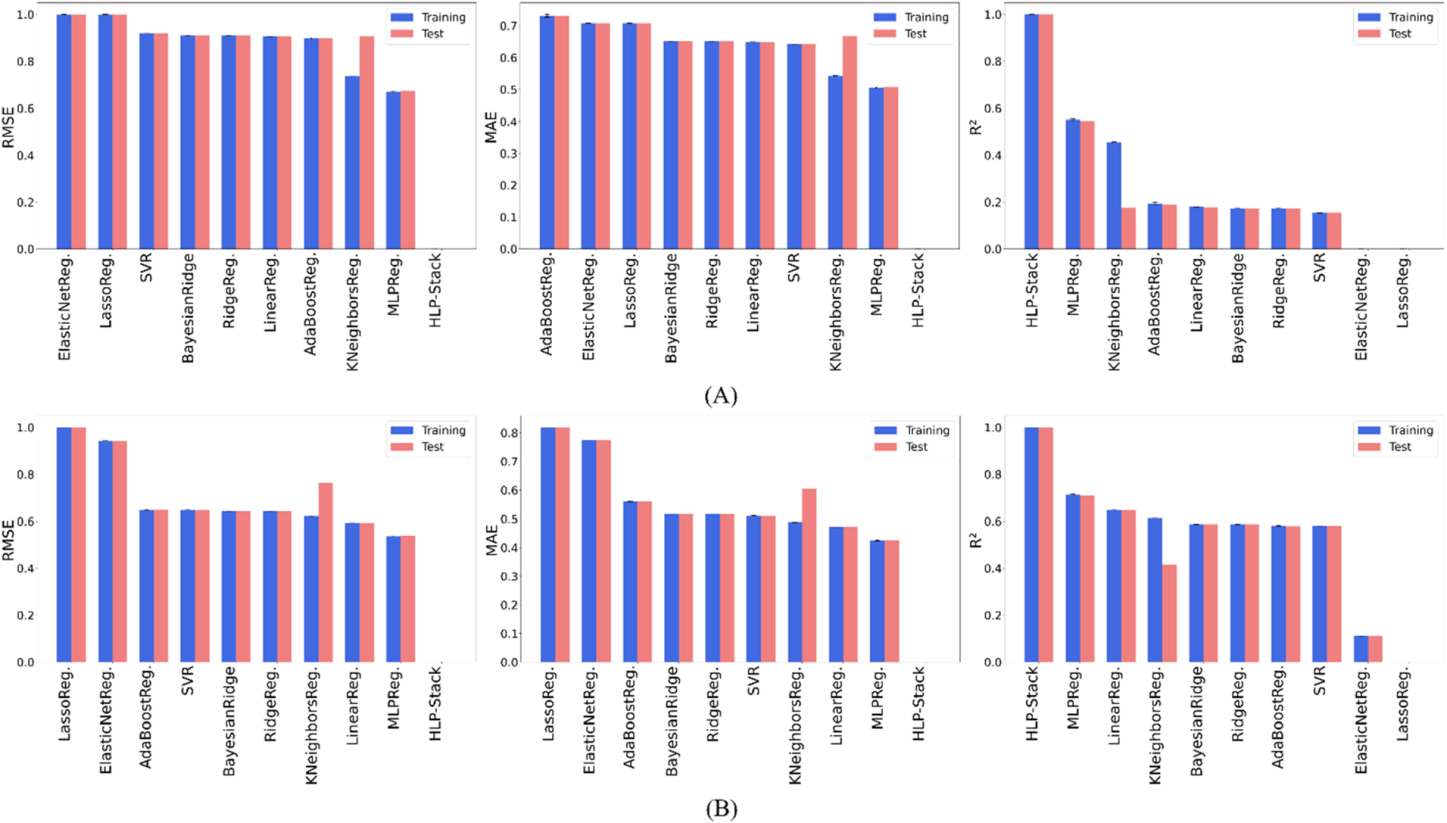
Comparative analysis of model performance for (A) HOMO and (B) LUMO prediction using 10-fold CV.

Fig. 10 presents a comparative performance of individual ML models and the HLP-Stack model using error bars to show variability across 10-fold Cross-Validation (CV). The metrics RMSE, MAE, and *R*^2^ are reported for both training and test datasets in the prediction of HOMO and LUMO energies. The HLP-Stack regressor consistently achieved the best performance across all statistical metrics for both targets, demonstrating its superior predictive capability. The MLP Regressor ranked second among individual models, delivering strong predictive performance. For HOMO energy, it achieved an average RMSE of 0.669 ± 0.008 *E*_h_ on the training set and 0.673 *E*_h_ on the test set, MAE values of 0.505 ± 0.009 *E*_h_ (training) and 0.507 *E*_h_ (test), and *R*^2^ values of 0.552 ± 0.011 (training) and 0.547 (test). Similarly, the MLP model demonstrated stable performance with average RMSE values of 0.537 ± 0.007 *E*_h_ (training) and 0.539 *E*_h_ (test), MAE values of 0.425 ± 0.007 *E*_h_ (training) and 0.426 *E*_h_ (test), and *R*^2^ values of 0.712 ± 0.008 (training) and 0.710 (test) for LUMO energy. These results highlight the robustness of the HLP-Stack model, which effectively integrates multiple regressors to achieve optimal accuracy. Detailed numerical results and fold-wise performance values for all models across all metrics are presented in the SI (Tables S2–S7).

### Analysis of HOMO–LUMO gap correlations for actual and predicted data

**Fig. 11:**
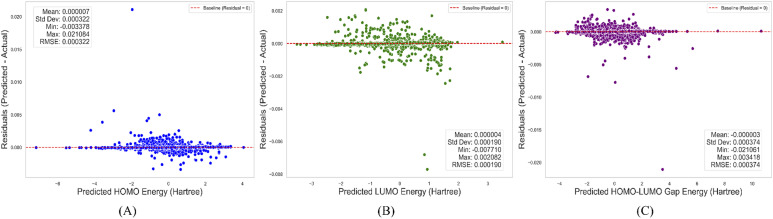
Residuals *vs.* predicted values for (A) HOMO, (B) LUMO, and (C) HOMO–LUMO gap on the test data.

We merged the actual and predicted values from separate test dataset evaluations to assess the reliability of the HLP-Stack model in predicting the HOMO and LUMO energies. The HOMO–LUMO energy gap was subsequently computed for both actual and predicted values. Pearson correlation coefficients were then calculated to analyse the relationships between the HOMO and LUMO energies, and the gap of between them. These correlations were assessed for both actual and predicted values to evaluate the model's predictive performance. Fig. 11 demonstrates these correlations, demonstrating that the predictive model effectively captures the expected inverse and direct correlations for HOMO and LUMO, respectively. The strong alignment between the actual and predicted values indicates that the model accurately preserves the physical relationships between HOMO and LUMO energy levels. The near-identical correlation values in the predicted data suggest that the model successfully learns and generalizes trends in the electronic structure.

### Analysis of residuals for the HOMO, LUMO, and HOMO–LUMO gap prediction

The HOMO–LUMO gap, which represents differences between predicted and actual values, was used to assess the accuracy and generalization ability of the model. To quantify the residuals further, we computed several statistical measures, including the mean, standard deviation (SD), minimum, maximum, and RMSE for each set of residuals. A summary of the residual statistics for the predictions is presented in Table 5. Residual analysis for the HOMO, LUMO, and HOMO–LUMO gap predictions revealed that the model performs well for all three properties. The markedly small mean residuals (close to zero) and low RMSE values indicate accurate predictions.

**Table 5:** Residual statistics for the predictions on the test set

Metric	HOMO (*E*_h_)	LUMO (*E*_h_)	HOMO–LUMO gap (*E*_h_)
Mean	7.00 × 10^−6^	4.00 × 10^−6^	−3.00 × 10^−6^
Std. Dev.	3.22 × 10^−4^	1.90 × 10^−4^	3.74 × 10^−4^
Min	−3.38 × 10^−3^	−7.71 × 10^−3^	−2.11 × 10^−2^
Max	2.11 × 10^−2^	2.08 × 10^−3^	3.42 × 10^−3^
RMSE	3.22 × 10^−4^	1.90 × 10^−4^	3.74 × 10^−4^

**Fig. 12:**
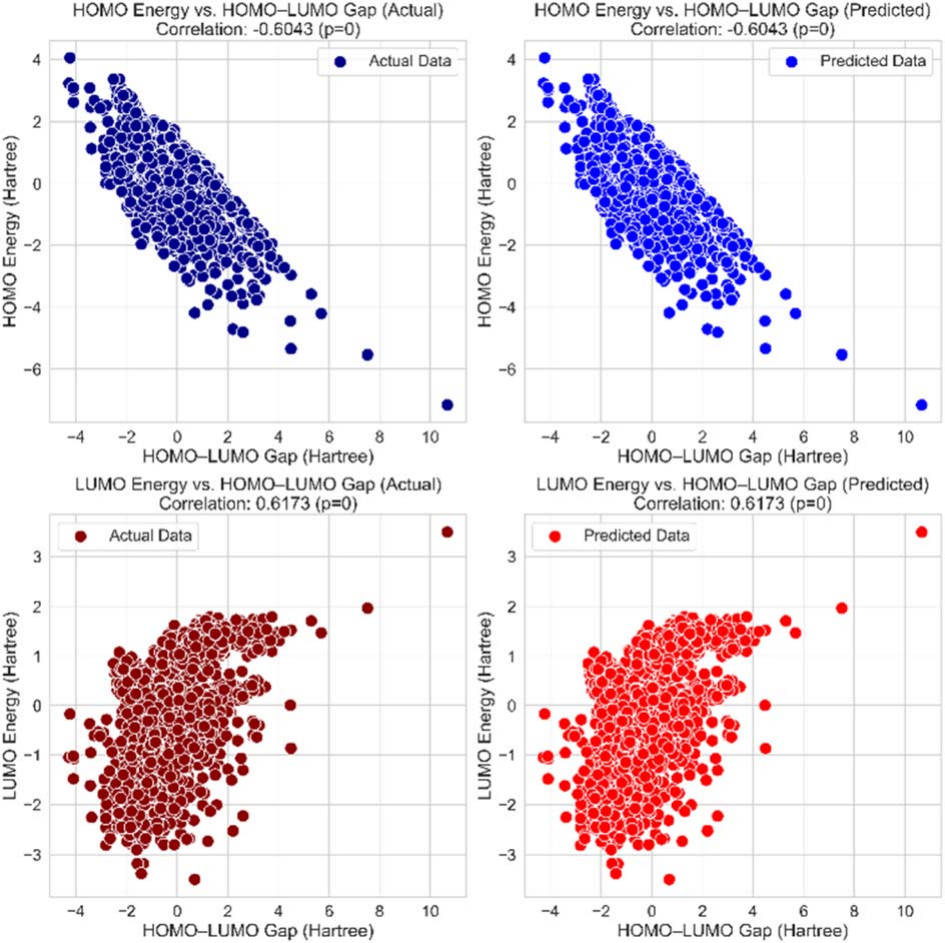
HOMO–LUMO gap correlations for actual and predicted test data.

The standard deviations were also small, suggesting consistency in the model's performance. However, larger deviations were occasionally observed, particularly for the HOMO–LUMO gap predictions, which exhibit the highest range and variability. The distribution of residuals against the predicted values is illustrated in Fig. 12. The scatter plots for HOMO, LUMO, and the HOMO–LUMO gap residuals show that the residuals are distributed around zero, suggesting an absence of significant bias in the model's predictions. Although the presence of a few larger residuals suggests that the model could be improved in specific regions of the feature space, the plots indicate that it is functioning well.

## Conclusions

In this study, we developed a stacking ensemble ML regressor to predict HOMO and LUMO energy values using molecular descriptors from the QM9 dataset. We achieved superior predictive accuracy by combining 2D molecular properties from RDKit and 3D quantum mechanical descriptors from DFT calculations. The SelectKBest algorithm, using mutual information regression, identified the most relevant features. Furthermore, the stacking ensemble model outperformed nine state-of-the-art ML models across all statistical metrics.

Correlation analysis between descriptors and target properties confirmed that no feature trivially encoded HOMO or LUMO energies, ensuring the absence of data leakage or redundant dependencies. The observed moderate correlation such as those between HOMO and molecular polarizability, and between LUMO and dipole moment, reflect physically meaningful relationships rather than direct encodings. This confirms that the models learn non-trivial, chemically interpretable patterns linking molecular structure to orbital energies. We employed the SHAP Tree Explainer to interpret feature importance, revealing key molecular properties affecting HOMO and LUMO energies. Additionally, our analyses of molecular topology and functional groups highlighted trends in aromaticity, ring structures, and their impact on electronic behaviour. The HOMO–LUMO gap analysis further demonstrated how the molecular framework and functionalization affect electronic properties.

Our findings confirm that the proposed model maintains the expected physical correlations between HOMO and LUMO energies and the HOMO–LUMO gap. The model demonstrates a robust ability to capture the underlying relationships in the energy data, as indicated by the strong correlation between actual and predicted values. Residual analysis suggests that the model is effective in predicting HOMO, LUMO, and the HOMO–LUMO gap with minimal bias and small prediction errors. Overall, this study validates the effectiveness of ensemble learning models for quantum property prediction and provides insights into structure–property relationships within the QM9 dataset. Future work could extend this approach to other datasets and alternative molecular representations for enhanced predictive modelling.

## Author contributions

Omid Mahmoudi: data curation, methodology, writing – original draft. Mi-hyun Kim: investigation, conceptualization, writing – review & revision, supervision, resources, methodology, funding acquisition, project administration.

## Conflicts of interest

There are no conflicts to declare.

## Supplementary Material

RA-016-D5RA08007J-s001

## Data Availability

All codes, raw and processed data, and analysis scripts used in this study are available at our GitHub repository: https://github.com/college-of-pharmacy-gachon-university/HLP_STACK. Supplementary information (SI): the list of selected molecular descriptors used in model training, SHAP-based feature importance, and a systematic investigation of HOMO–LUMO gap trends with varying ring count and functional groups. Additionally, a summary of ML models, their performance metrics, and visualizations of representative molecular structures with trends in HOMO–LUMO gaps are provided. See DOI: https://doi.org/10.1039/d5ra08007j.
