# Supplementary material for: Predicting electronic properties of molecules: a stacking ensemble model for HOMO and LUMO energy estimation
Source: RSC Adv. 2026 Mar 4;16(14):12444–57. doi: 10.1039/d5ra08007j (PMC12959570; doi:10.1039/d5ra08007j)
Supplement: RA-016-D5RA08007J-s001 [file RA-016-D5RA08007J-s001.pdf]

Supplementary Information (SI) for RSC Advances.
This journal is © The Royal Society of Chemistry 2026

Predicting Electronic Properties of Molecules: A Stacking Ensemble Model for HOMO and LUMO Energy Estimation

Omid Mahmoudi, https://orcid.org/0000-0002-4842-473X*[a] and Mi-hyun Kim https://orcid.org/0000-0002-2718-5637[a]

[a] Department of Pharmacy, Gachon University, Incheon, South Korea. E-mail: omid@gachon.ac.kr, kmh0515@gachon.ac.kr

Contents

Refined Molecular Descriptors
Table S1 Selected Features using Mutual Information Regression Method 4

Correlation Analysis and Feature Independence Assessment
Fig. S1 Correlation analysis of all extracted features: (A) correlation matrix showing pairwise relationships among features; (B) heatmap highlighting feature pairs with strong correlations (|r| > 0.8) 6
Table S2 Performance of RF Models Before and After Removal of Strongly Correlated Features 7

Positive and Negative SHAP Values
Fig. S2 Top 10 Features with Positive SHAP Values for HOMO 8
Fig. S3 Top 10 Features with Negative SHAP Values for LUMO 8

Variation of Mean HOMO-LUMO Gap with Carbon Count Across Non-PAH and PAH Structures
Fig. S4 Mean HOMO-LUMO Gap vs. Carbon Count for Non-PAH (0 Aromatic Rings, 1 Total Rings) and Various Functional Groups 9
Fig. S5 Mean HOMO-LUMO Gap vs. Carbon Count for Non-PAH (0 Aromatic Rings, 2 Total Rings) and Various Functional Groups 9
Fig. S6 Mean HOMO-LUMO Gap vs. Carbon Count for Non-PAH (0 Aromatic Rings, 3 Total Rings) and Various Functional Groups 10
Fig. S7 Mean HOMO-LUMO Gap vs. Carbon Count for Non-PAH (0 Aromatic Rings, 4 Total Rings) and Various Functional Groups 10
Fig. S8 Mean HOMO-LUMO Gap vs. Carbon Count for Non-PAH (0 Aromatic Rings, 5 Total Rings) and Various Functional Groups 11
Fig. S9 Mean HOMO-LUMO Gap vs. Carbon Count for Non-PAH (0 Aromatic Rings, 6 Total Rings) and Various Functional Groups 11
Fig. S10 Mean HOMO-LUMO Gap vs. Carbon Count for Non-PAH (0 Aromatic Rings, 7 Total Rings) and Various Functional Groups 12
Fig. S11 Mean HOMO-LUMO Gap vs. Carbon Count for Non-PAH (0 Aromatic Rings, 8 Total Rings) and Various Functional Groups 12
Fig. S12 Mean HOMO-LUMO Gap vs. Carbon Count for Non-PAH (1 Aromatic Rings, 2 Total Rings) and Various Functional Groups 13
Fig. S13 Mean HOMO-LUMO Gap vs. Carbon Count for Non-PAH (1 Aromatic Rings, 3 Total Rings) and Various Functional Groups 14
Fig. S14 Mean HOMO-LUMO Gap vs. Carbon Count for Non-PAH (1 Aromatic Rings, 4 Total Rings) and Various Functional Groups 14
Fig. S15 Mean HOMO-LUMO Gap vs. Carbon Count for PAH-1 (Single Benzene Single Aromatic Rings) and Various Functional Groups 14

Visualization of Functional Group Effects on HOMO-LUMO Gaps
Fig. S16 Visualization of HOMO-LUMO Gaps with Annotated Molecular Structures for Non-PAH (0 Aromatic Rings, 1 Total Rings) 15
Fig. S17 Visualization of HOMO-LUMO Gaps with Annotated Molecular Structures for Non-PAH (0 Aromatic Rings, 2 Total Rings) 16
Fig. S18 Visualization of HOMO-LUMO Gaps with Annotated Molecular Structures for Non-PAH 17

(0 Aromatic Rings, 3 Total Rings)
Fig. S19 Visualization of HOMO-LUMO Gaps with Annotated Molecular Structures for Non-PAH (0 Aromatic Rings, 4 Total Rings) 18
Fig. S20 Visualization of HOMO-LUMO Gaps with Annotated Molecular Structures for Non-PAH (0 Aromatic Rings, 5 Total Rings) 19
Fig. S21 Visualization of HOMO-LUMO Gaps with Annotated Molecular Structures for Non-PAH (0 Aromatic Rings, 6 Total Rings) 20
Fig. S22 Visualization of HOMO-LUMO Gaps with Annotated Molecular Structures for Non-PAH (0 Aromatic Rings, 7 Total Rings) 21
Fig. S23 Visualization of HOMO-LUMO Gaps with Annotated Molecular Structures for Non-PAH (0 Aromatic Rings, 8 Total Rings) 21
Fig. S24 Visualization of HOMO-LUMO Gaps with Annotated Molecular Structures for Non-PAH (1 Aromatic Rings, 1 Total Rings) 22
Fig. S25 Visualization of HOMO-LUMO Gaps with Annotated Molecular Structures for Non-PAH (1 Aromatic Rings, 2 Total Rings) 23
Fig. S26 Visualization of HOMO-LUMO Gaps with Annotated Molecular Structures for Non-PAH (1 Aromatic Rings, 3 Total Rings) 24
Fig. S27 Visualization of HOMO-LUMO Gaps with Annotated Molecular Structures for Non-PAH (1 Aromatic Rings, 4 Total Rings) 24
Fig. S28 Visualization of HOMO-LUMO Gaps with Annotated Molecular Structures for Non-PAH (2 Aromatic Rings, 2 Total Rings) 24
Fig. S29 Visualization of HOMO-LUMO Gaps with Annotated Molecular Structures for PAH-1 (Single Benzene 1 Aromatic Rings) 25
Fig. S30 Visualization of HOMO-LUMO Gaps with Annotated Molecular Structures for PAH-1 (Single Benzene 2 Aromatic Rings) 25

Machine Learning Models

Adaptive Boosting Regressor (AdaBoostRegressor) 26
Bayesian Ridge Regression (BRR) 26
Elastic Net Regression (ENet) 26
K-Nearest Neighbors Regressor (KNNR) 26
Linear Regression (LR) 26
Lasso Regression (Least Absolute Shrinkage and Selection Operator, Lasso) 26
Multi-Layer Perceptron Regressor (MLPR) 26
Ridge Regression (RR) 26
Support Vector Regression (SVR) 26
Random Forest (RF) 26
Extreme Gradient Boosting (XGBoost, XGB) 26
Extra Trees (ET) 27
Gradient Boosting (GB) 27

Results for 10-Fold Cross-Validation

Table S3 RMSE Results for 10-Fold Cross-Validation: HOMO Energy 28
Table S4 RMSE Results for 10-Fold Cross-Validation: LUMO Energy 28
Table S5 MAE Results for 10-Fold Cross-Validation: HOMO Energy 29
Table S6 MAE Results for 10-Fold Cross-Validation: LUMO Energy 29
Table S7 R^2 Results for 10-Fold Cross-Validation: HOMO Energy 30
Table S8 R^2 Results for 10-Fold Cross-Validation: LUMO Energy 30

Refined Molecular Descriptors

Table S1 Selected Features using Mutual Information Regression Method.

No.	Feat. Idx.	Descriptor	Description
1	Ft0	fr_amidine	Describes the presence of an amidine group (–C(=NH)–NH2), which can contribute to nucleophilicity and hydrogen bonding.
2	Ft1	NumHAcceptors	The number of hydrogen bond acceptors, typically electronegative atoms like oxygen and nitrogen that can accept hydrogen bonds from donors.
3	Ft2	fr_urea	Indicates the presence of a urea functional group (–NH–CO–NH–), commonly seen in drugs and bioactive molecules due to its hydrogen bonding properties.
4	Ft3	Chi3n	A topological index based on the third-order neighbors, providing information on molecular branching and structure complexity.
5	Ft4	Chi1n	A topological index related to the first-order neighbors, indicating the degree of connectivity of atoms within the molecule.
6	Ft5	zpve	Zero-point vibrational energy, often used in quantum mechanical calculations, indicating the lowest energy state of the molecule.
7	Ft6	Ipc	Ionization potential, representing the energy required to remove an electron, influencing a molecule's reactivity.
8	Ft7	Chi1v	A topological index similar to Chi1n but considering the molecular valence structure, often used for predicting molecular interactions.
9	Ft8	SMR_VSA2	Surface area related to the molecular surface descriptor SMR, capturing the extent of molecular interactions and solvent accessibility.
10	Ft9	fr_nitroso	Indicates the presence of a nitroso group (–NO), which is often involved in redox reactions and can influence reactivity.
11	Ft10	fr_phenol	Presence of a phenolic group (–OH attached to an aromatic ring), which is important for hydrogen bonding, and also contributes to antioxidant properties.
12	Ft11	BCUT2D_MRLOW	A descriptor related to the molecular weight distribution of a molecule, focusing on low-molecular-weight compounds, and used to assess the size of the molecule.
13	Ft12	VSA_EState4	A descriptor based on the molecular electrostatic potential surface area, indicating the polarity and distribution of charges within the molecule.
14	Ft13	PEOE_VSA2	Represents the electrostatic surface area associated with polar and non-polar regions, used for assessing solubility and interaction potential.
15	Ft14	fr_unbrch_alkane	Indicates the presence of a non-branched alkane group, which is typically hydrophobic and contributes to molecular stability.
16	Ft15	SASA (Å²)	Solvent accessible surface area, representing the surface area of a molecule accessible to solvent molecules, often used to predict solubility and reactivity.
17	Ft16	PEOE_VSA5	Similar to PEOE_VSA2 but focused on a different region of the molecular electrostatic potential surface area.
18	Ft17	SlogP_VSA1	A descriptor related to the solvent-accessible surface area and the octanol-water partition coefficient (LogP), which helps estimate lipophilicity.
19	Ft18	BertzCT	A topological descriptor capturing information on the complexity and branching of the molecular structure, used in quantitative structure-activity relationships (QSAR).
20	Ft19	fr_amide	Indicates the presence of an amide group (–CO–NH2), commonly involved in protein binding, and is important for hydrogen bonding.
21	Ft20	mu	The dipole moment of the molecule, indicating its polarity and how the molecule might interact with electric fields.
22	Ft21	BalabanJ	A topological descriptor related to the molecular complexity and cyclic structure, often used to estimate chemical reactivity.
23	Ft22	Steric Hindrance	Describes the spatial arrangement of atoms and the resulting repulsive forces that affect a molecule's ability to undergo reactions.
24	Ft23	fr_priamide	A pyrimidine-derived amide group, contributing to the molecule's ability to interact with other compounds via hydrogen bonds.
25	Ft24	fr_ester	Presence of an ester group (–COO–), which is common in esters and contributes to molecular solubility and reactivity.

Table S1 continued.

No.	Feat. Idx.	Descriptor	Description
26	Ft25	fr_C_O_noCOO	Indicates the presence of a carbon-oxygen bond without an ester group, often contributing to a molecule's polarity.
27	Ft26	fr_azo	Represents the presence of an azo group (–N=N–), which can affect reactivity, often involved in dye and pharmaceutical chemistry.
28	Ft27	fr_NH1	Presence of a primary amine group (–NH2), contributing to hydrogen bonding and reactivity.
29	Ft28	Chi3v	A topological index considering the third-order valence structure, similar to Chi3n but focusing on molecular valence.
30	Ft29	fr_para_hydroxylation	Indicates a para-hydroxylation, where a hydroxyl group is attached to an aromatic ring in the para position, contributing to molecular solubility and reactivity.
31	Ft30	EState_VSA2	A descriptor related to the electrostatic surface area of the molecule, indicating the extent of polarization.
32	Ft31	qed	The quantitative estimate of drug-likeness, representing how likely a molecule is to be drug-like based on its physicochemical properties.
33	Ft32	fr_COO2	Represents the presence of a carboxyl group (–COOH), involved in acid-base chemistry and hydrogen bonding.
34	Ft33	alpha	A molecular descriptor representing the polarizability of the molecule, contributing to its interaction with electric fields.
35	Ft34	r2	The square of the correlation coefficient, typically used to assess the quality of regression models or fit.
36	Ft35	fr_epoxide	Indicates the presence of an epoxide group (–O–), which is highly reactive and can participate in nucleophilic reactions.
37	Ft36	g298	The Gibbs free energy of the molecule at 298K, which provides insights into the thermodynamic stability of the compound.
38	Ft37	u0	The internal energy of the molecule at 0K, useful for thermodynamic assessments.
39	Ft38	PEOE_VSA11	Another electrostatic surface area descriptor, capturing different interactions within the molecule's electrostatic potential.
40	Ft39	fr_morpholine	Indicates the presence of a morpholine group (a six-membered ring containing oxygen and nitrogen), contributing to hydrogen bonding and potential reactivity.
41	Ft40	Kappa1	A topological descriptor that reflects the branching and size of the molecule, used to estimate molecular shape and complexity.
42	Ft41	u298	The internal energy of the molecule at 298K, which helps assess the molecule's thermodynamic behavior.
43	Ft42	EState_VSA7	A descriptor that measures the electrostatic surface area for specific regions of the molecule, contributing to its reactivity and solubility.
44	Ft43	MaxPartialCharge	The maximum partial charge on any atom within the molecule, indicating regions of high reactivity.
45	Ft44	cv	A descriptor that quantifies the variation in molecular properties or structure, used in assessing stability and diversity.
46	Ft45	h298	The enthalpy changes at 298K, which can help assess the heat content and stability of the molecule.
47	Ft46	BCUT2D_LOGPLOW	A molecular descriptor that combines molecular structure with LogP (octanol-water partition coefficient) to predict solubility and hydrophobicity.
48	Ft47	fr_hdrzone	Represents the presence of a hydrogen-bond donor zone, important for hydrogen bonding interactions.
49	Ft48	NumHDonors	The number of hydrogen bond donors (atoms like nitrogen or oxygen with hydrogen attached) that can form hydrogen bonds.
50	Ft49	Chi1	A topological index related to the first-order neighbors, indicating the extent of connectivity between atoms.
51	Ft50	fr_Ndealkylation1	Indicates the potential for a molecule to undergo N-dealkylation (removal of an alkyl group from a nitrogen atom), which can influence metabolism.

Correlation Analysis and Feature Independence Assessment

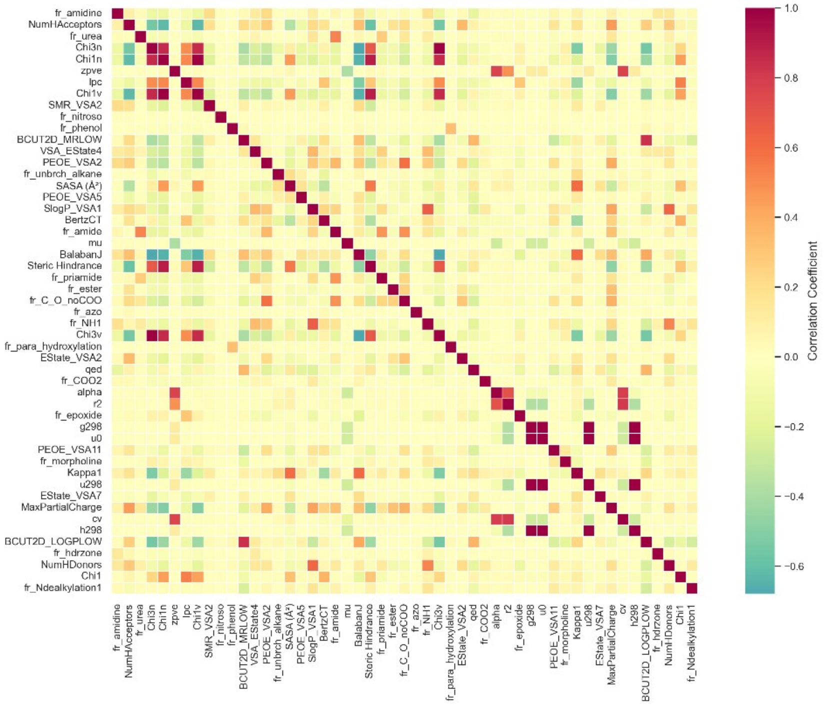

(A)

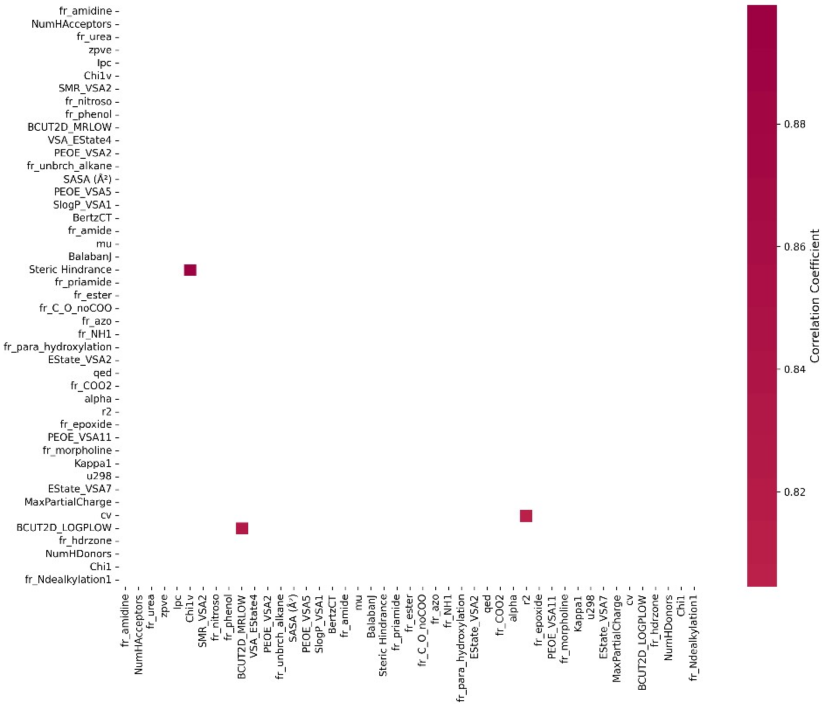

(B)

Fig. S1 Correlation analysis of all extracted features: (A) correlation matrix showing pairwise relationships among features; (B) heatmap highlighting feature pairs with strong correlations (|r| > 0.8).

Table S2 Performance comparison of Random Forest Regressor models trained for HOMO and LUMO energy prediction using the complete feature set (51 descriptors) and the correlation-reduced dataset. The nearly identical R^2, RMSE, and MAE values confirm that the removal of strongly correlated descriptors (g298, u0, h298, Chi3n, Chi1n, and Chi3v) had a negligible impact on predictive performance, indicating minimal redundancy and strong feature independence.

Feature Set	Target	R^2	RMSE (E_h)	MAE (E_h)
Full (51 Descriptors)	HOMO	1.0	1.32e-04	6.26e-06
	LUMO	1.0	7.64e-05	3.31e-06
Correlation--Filtered	HOMO	1.0	1.37e-04	7.23e-06
	LUMO	1.0	7.60e-05	3.02e-06

Positive and Negative SHAP Values

The positive and negative SHAP values for 10 most important features are illustrated in Fig. S1 and S2.

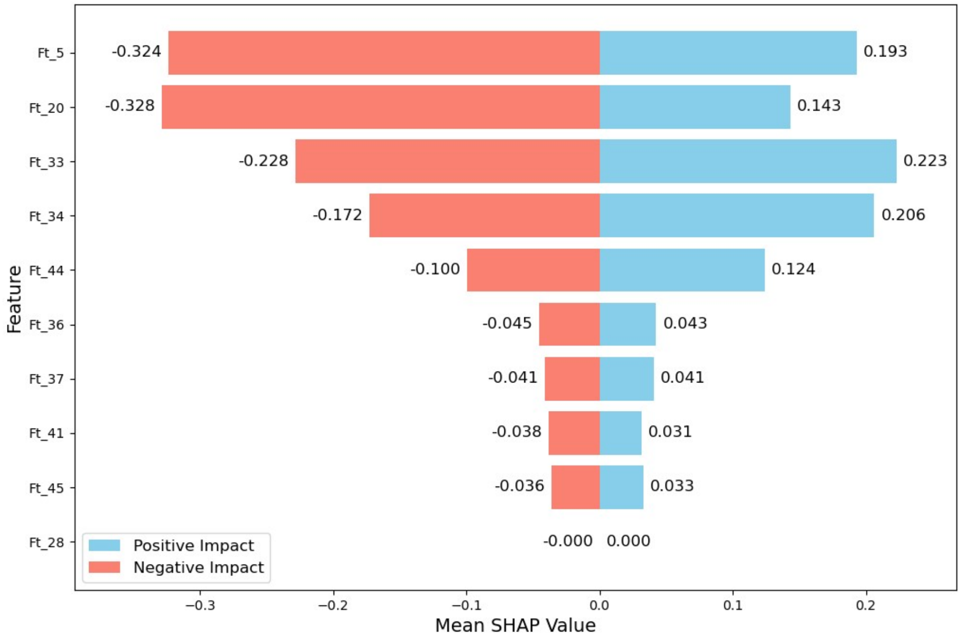

Fig. S2 Top 10 Features with Positive and Negative SHAP Values for HOMO.

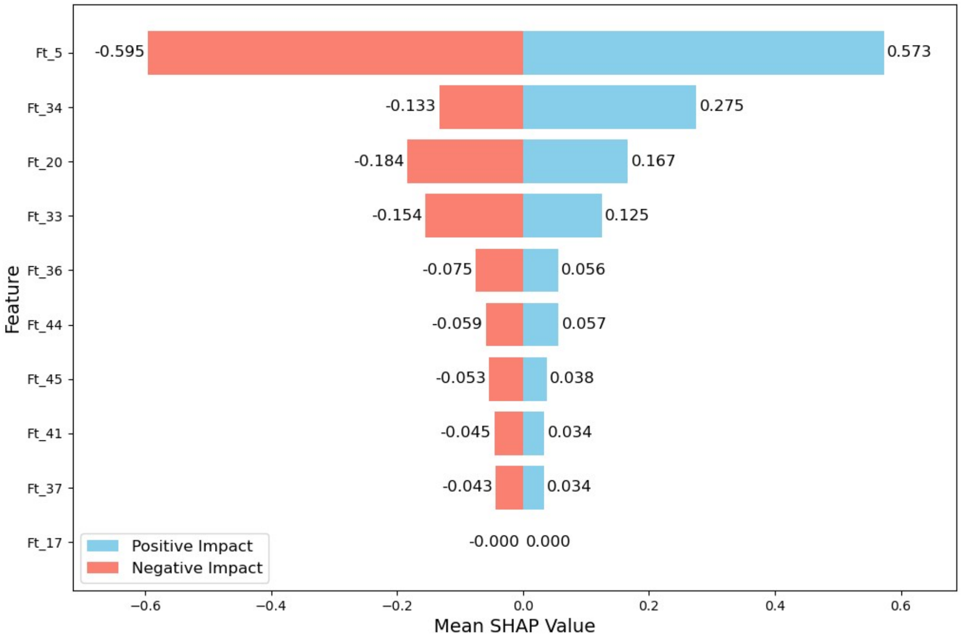

Fig. S3 Top 10 Features with Positive and Negative SHAP Values for LUMO.

Variation of Mean HOMO-LUMO Gap with Carbon Count Across Non-PAH and PAH Structures

This Fig. set illustrates the relationship between the mean HOMO-LUMO gap and the number of carbon atoms in molecules featuring diverse ring systems and functional groups. Each figure provides insights into how increasing carbon count and ring complexity influence electronic structure, particularly for different functional groups within each category. These trends can aid in understanding how molecular topology and functionalization affect electronic properties relevant to materials and molecular design.

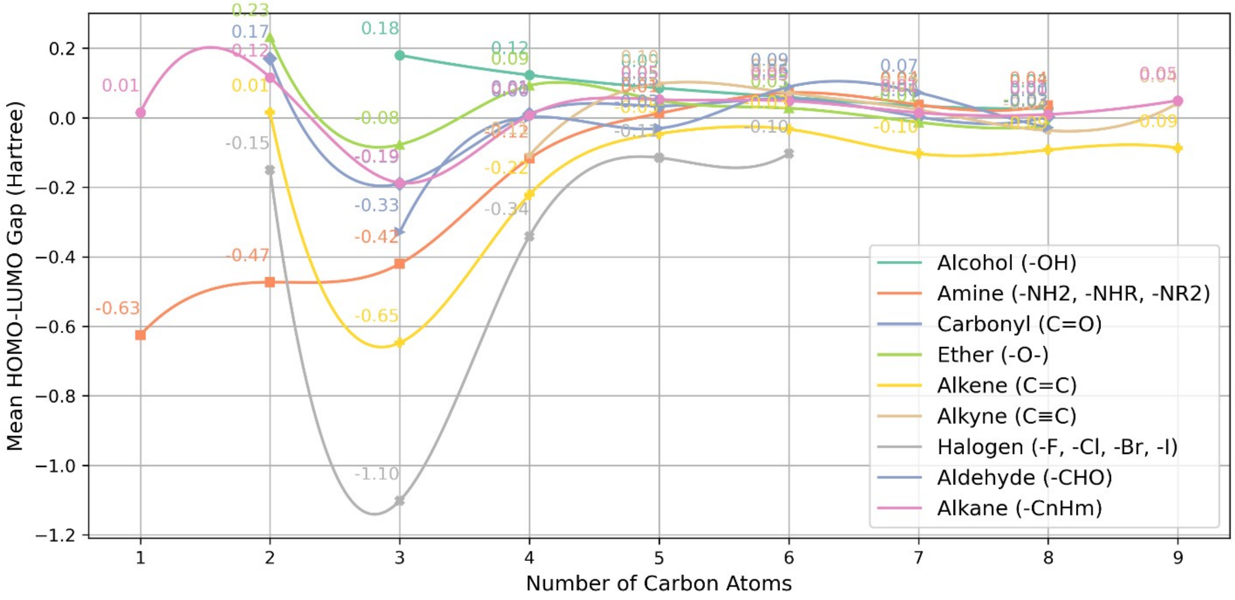

Fig. S4 Mean HOMO-LUMO Gap vs. Carbon Count for Non-PAH (0 Aromatic Rings, 1 Total Rings) and Various Functional Groups.

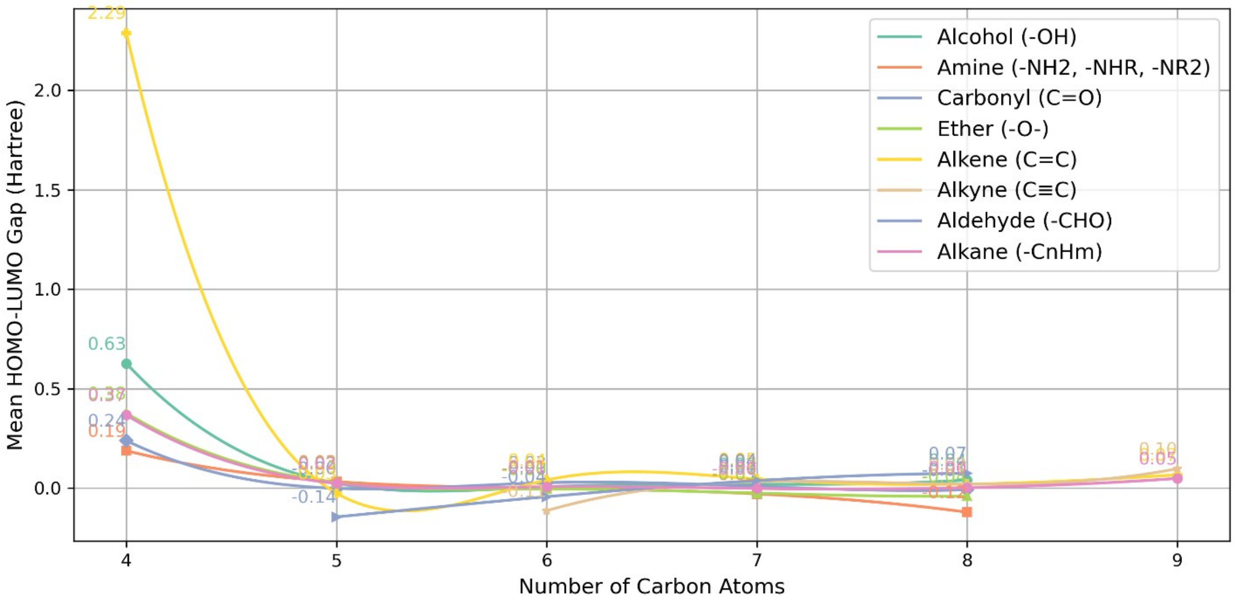

Fig. S5 Mean HOMO-LUMO Gap vs. Carbon Count for Non-PAH (0 Aromatic Rings, 2 Total Rings) and Various Functional Groups.

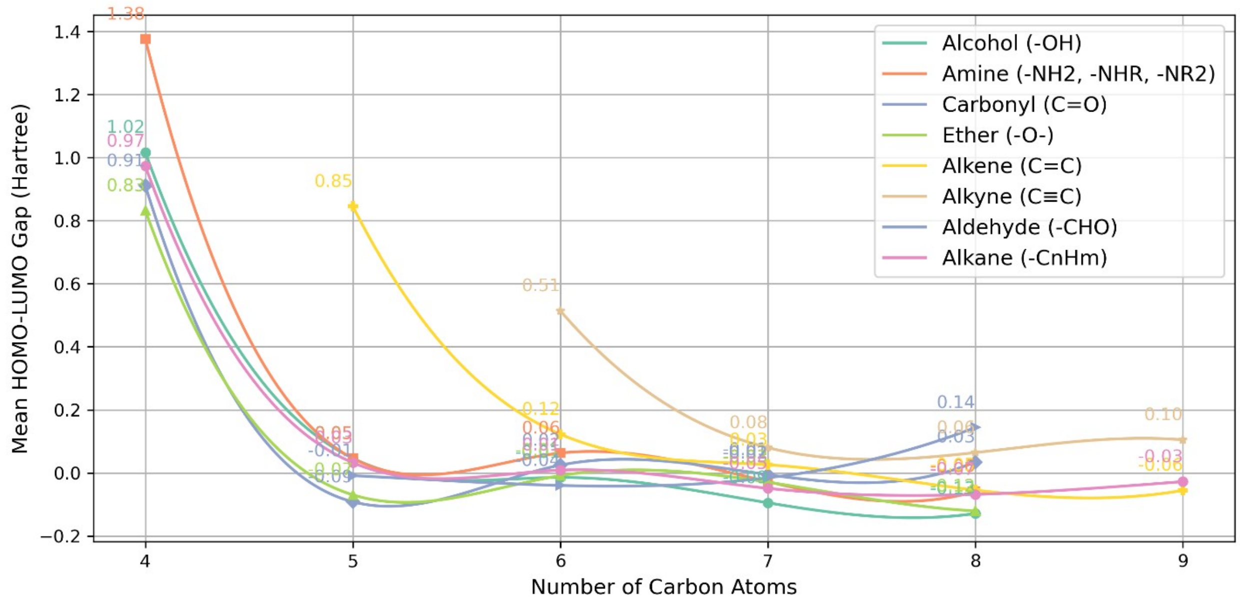

Fig. S6 Mean HOMO-LUMO Gap vs. Carbon Count for Non-PAH (0 Aromatic Rings, 3 Total Rings) and Various Functional Groups.

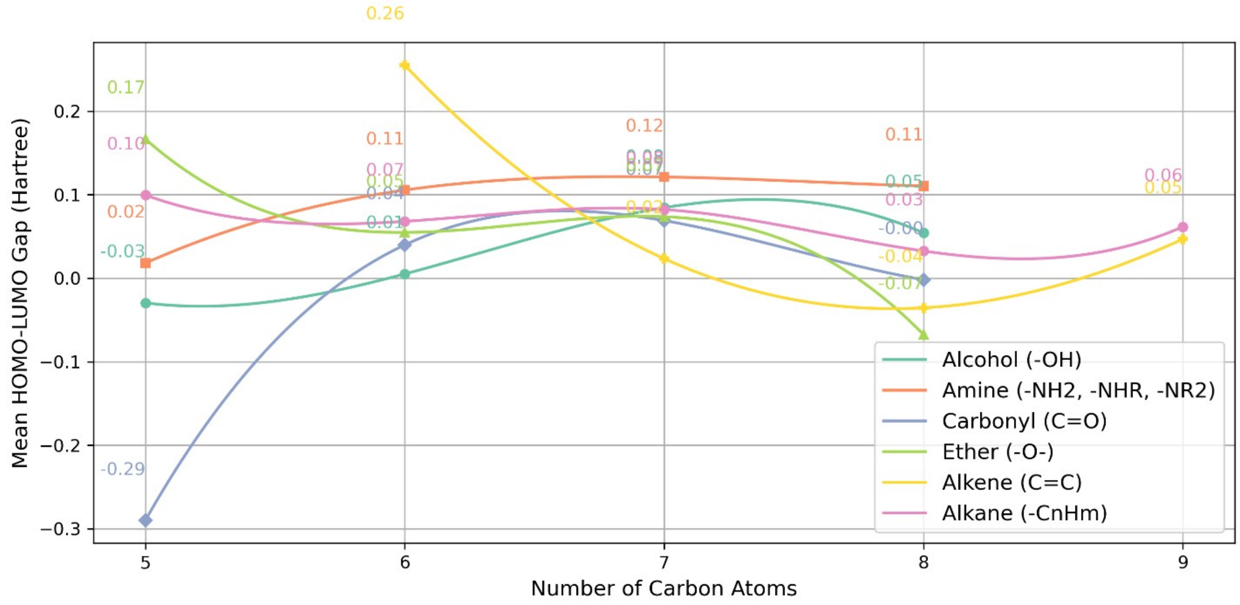

Fig. S7 Mean HOMO-LUMO Gap vs. Carbon Count for Non-PAH (0 Aromatic Rings, 4 Total Rings) and Various Functional Groups.

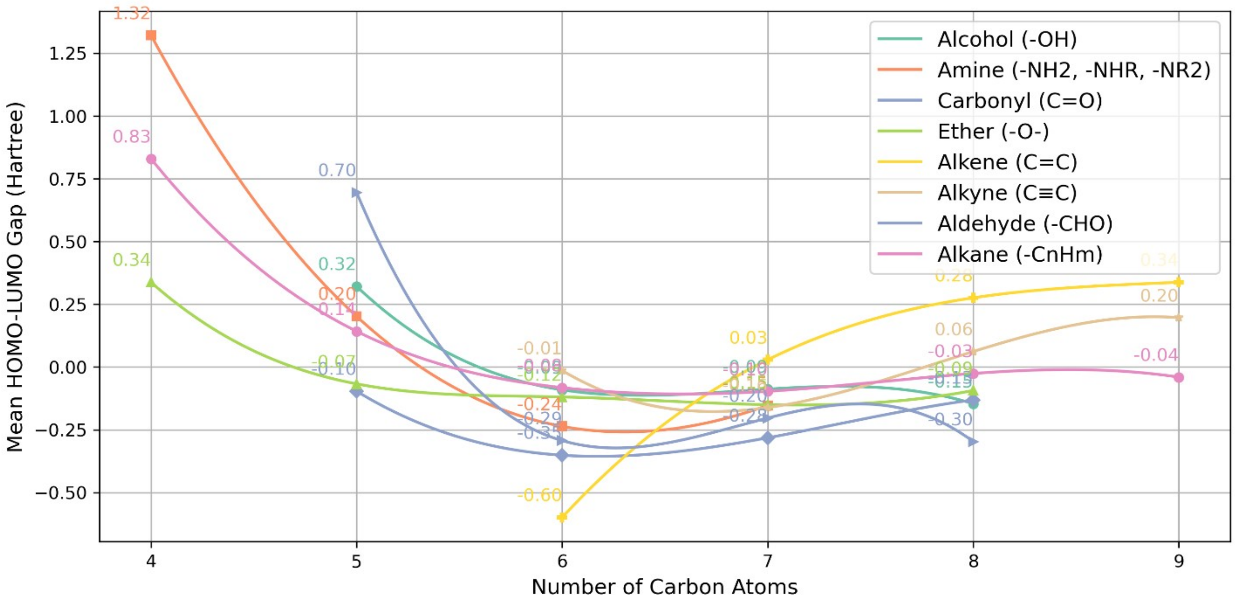

Fig. S8 Mean HOMO-LUMO Gap vs. Carbon Count for Non-PAH (0 Aromatic Rings, 5 Total Rings) and Various Functional Groups.

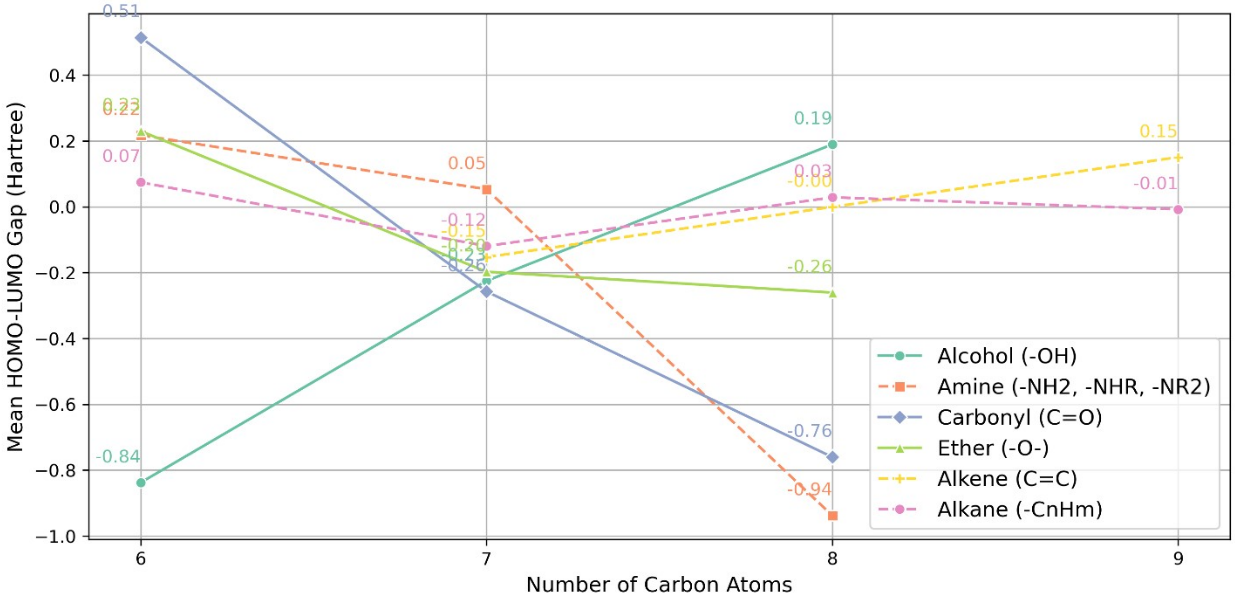

Fig. S9 Mean HOMO-LUMO Gap vs. Carbon Count for Non-PAH (0 Aromatic Rings, 6 Total Rings) and Various Functional Groups.

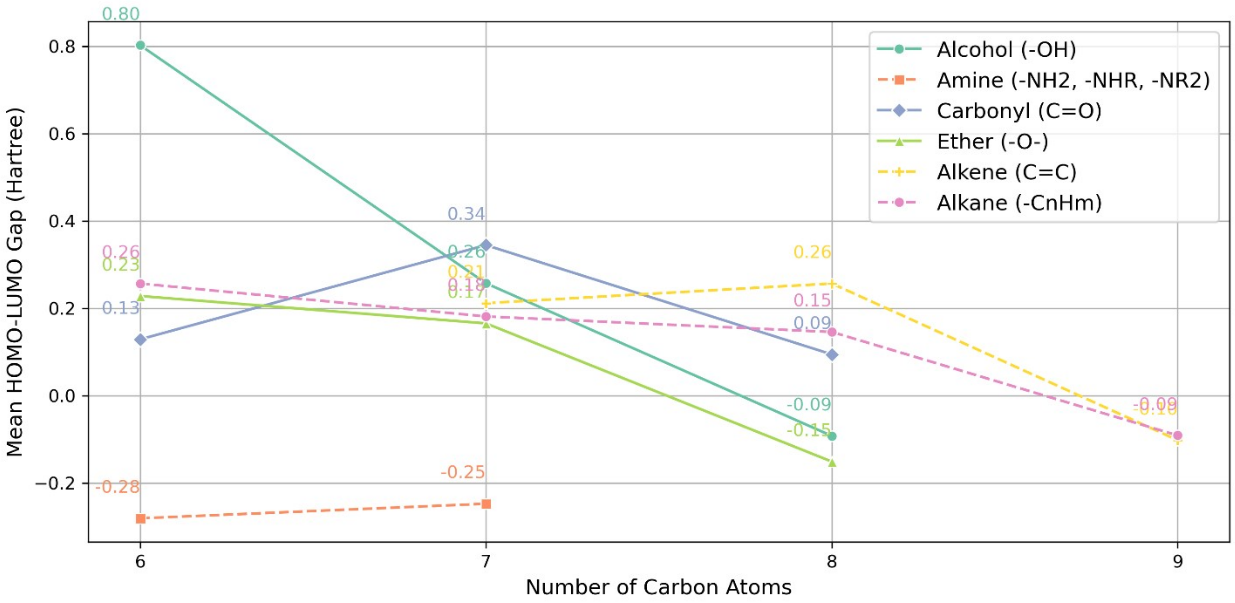

Fig. S10 Mean HOMO-LUMO Gap vs. Carbon Count for Non-PAH (0 Aromatic Rings, 7 Total Rings) and Various Functional Groups.

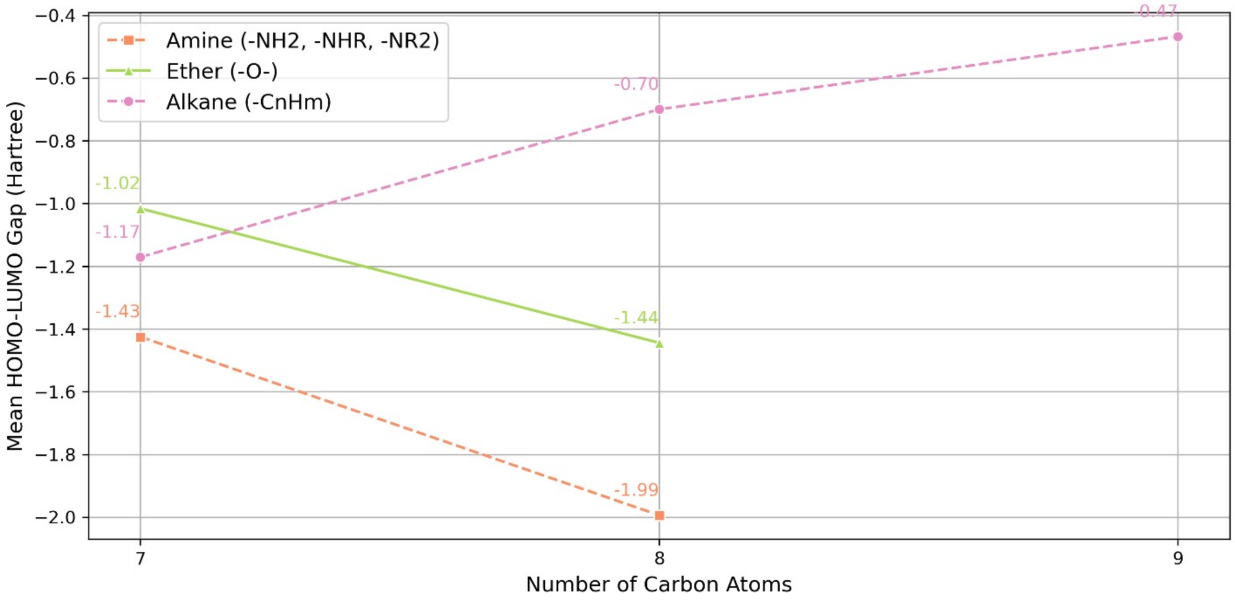

Fig. S11 Mean HOMO-LUMO Gap vs. Carbon Count for Non-PAH (0 Aromatic Rings, 8 Total Rings) and Various Functional Groups.

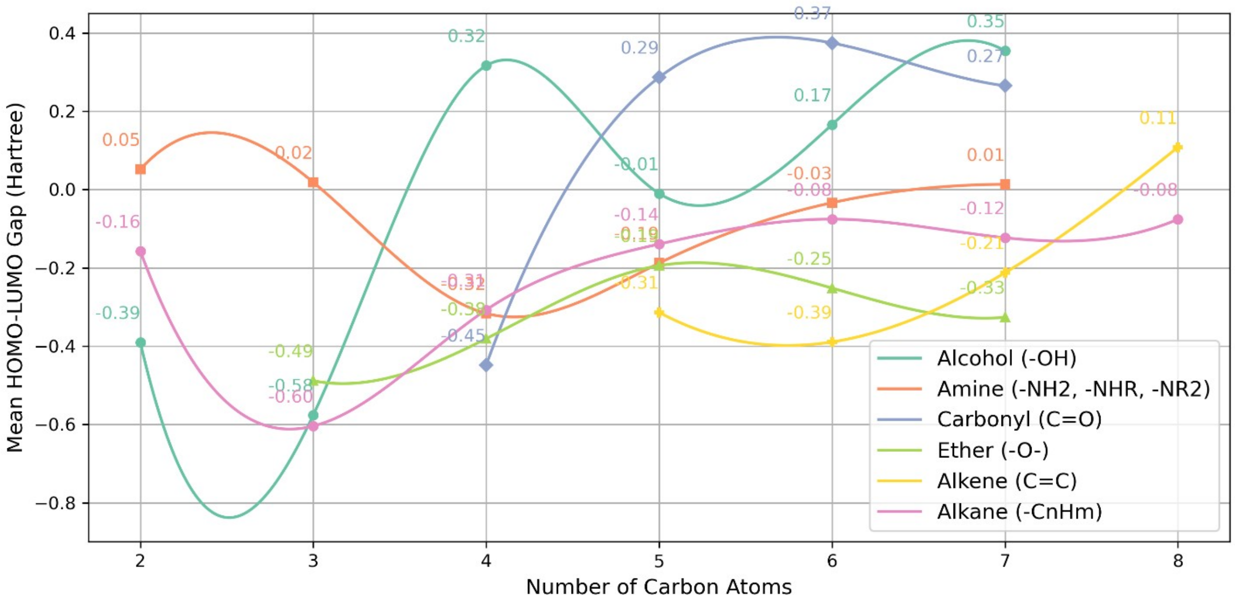

Fig. S12 Mean HOMO-LUMO Gap vs. Carbon Count for Non-PAH (1 Aromatic Rings, 2 Total Rings) and Various Functional Groups.

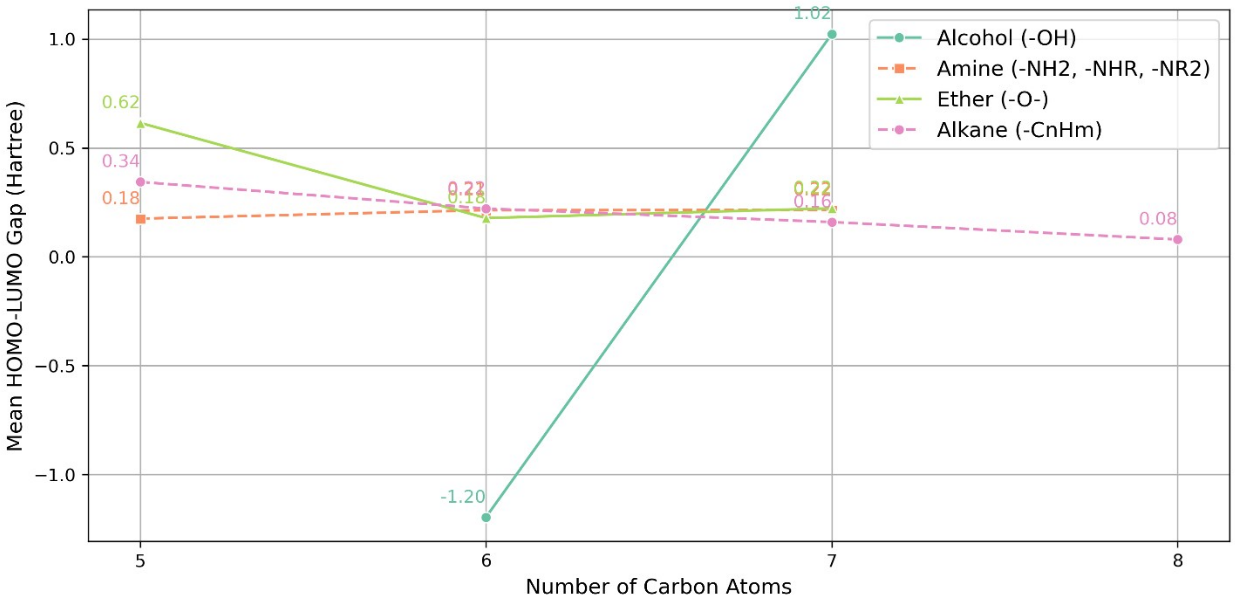

Fig. S13 Mean HOMO-LUMO Gap vs. Carbon Count for Non-PAH (1 Aromatic Rings, 3 Total Rings) and Various Functional Groups.

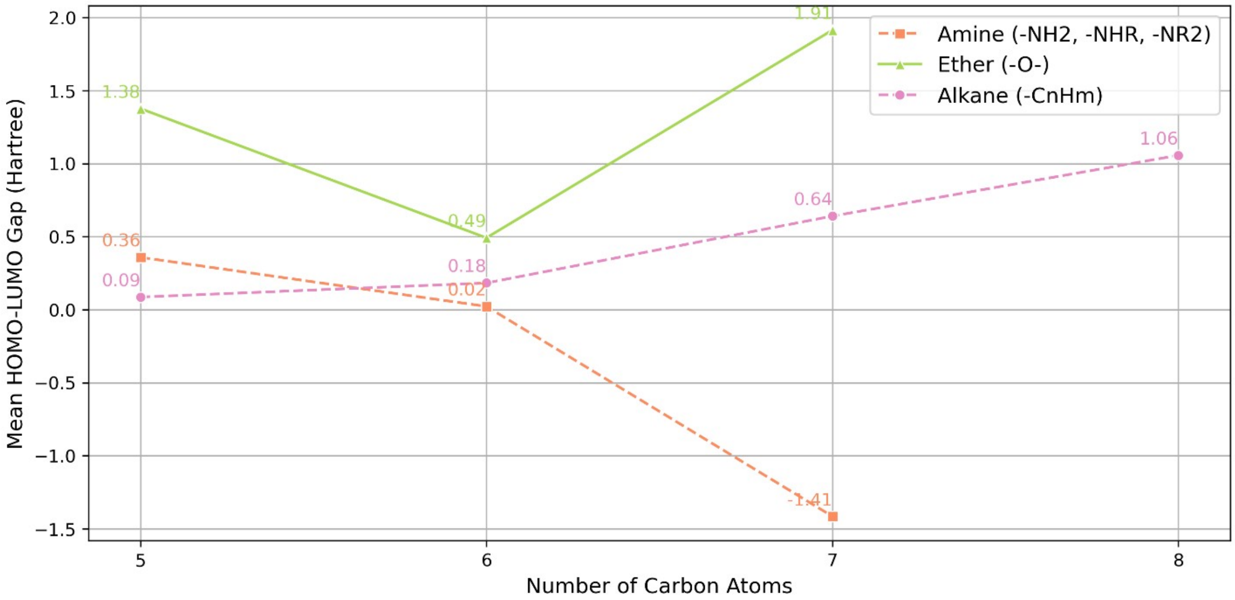

Fig. S14 Mean HOMO-LUMO Gap vs. Carbon Count for Non-PAH (1 Aromatic Rings, 4 Total Rings) and Various Functional Groups.

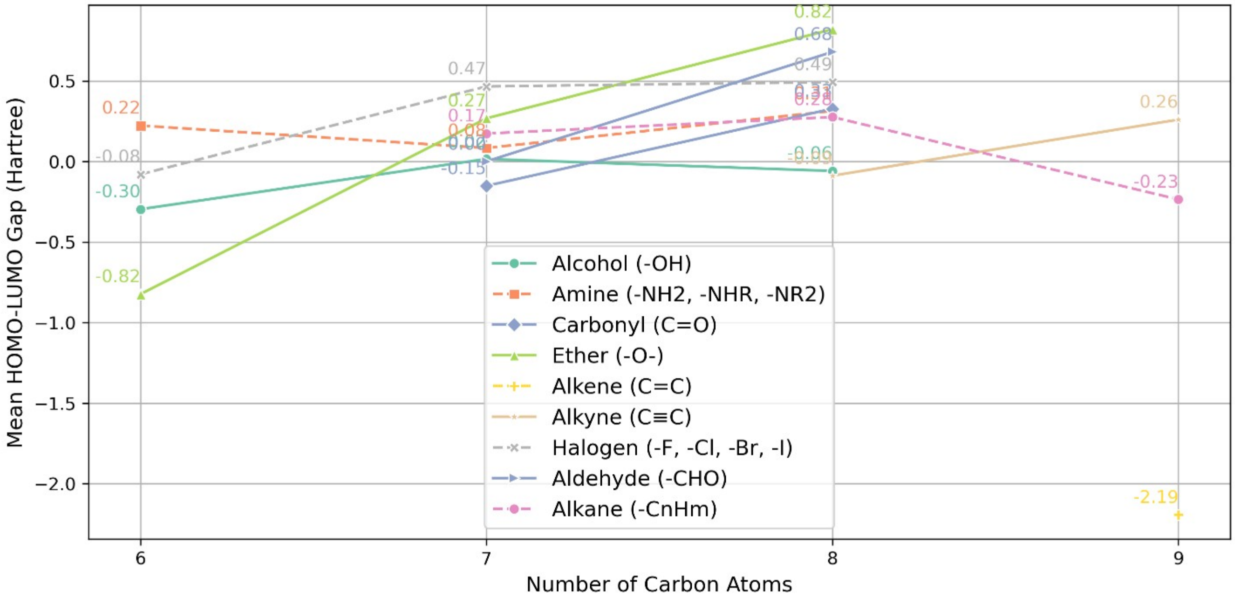

Fig. S15 Mean HOMO-LUMO Gap vs. Carbon Count for PAH-1 (Single Benzene Single Aromatic Rings) and Various Functional Groups.

Visualization of Functional Group Effects on HOMO-LUMO Gaps

This section presents a comprehensive visualization of the HOMO-LUMO energy gaps across various functional groups and molecular frameworks. Each figure illustrates molecules categorized by the number of aromatic and total rings, with annotated molecular structures highlighting how different functional groups influence electronic properties.

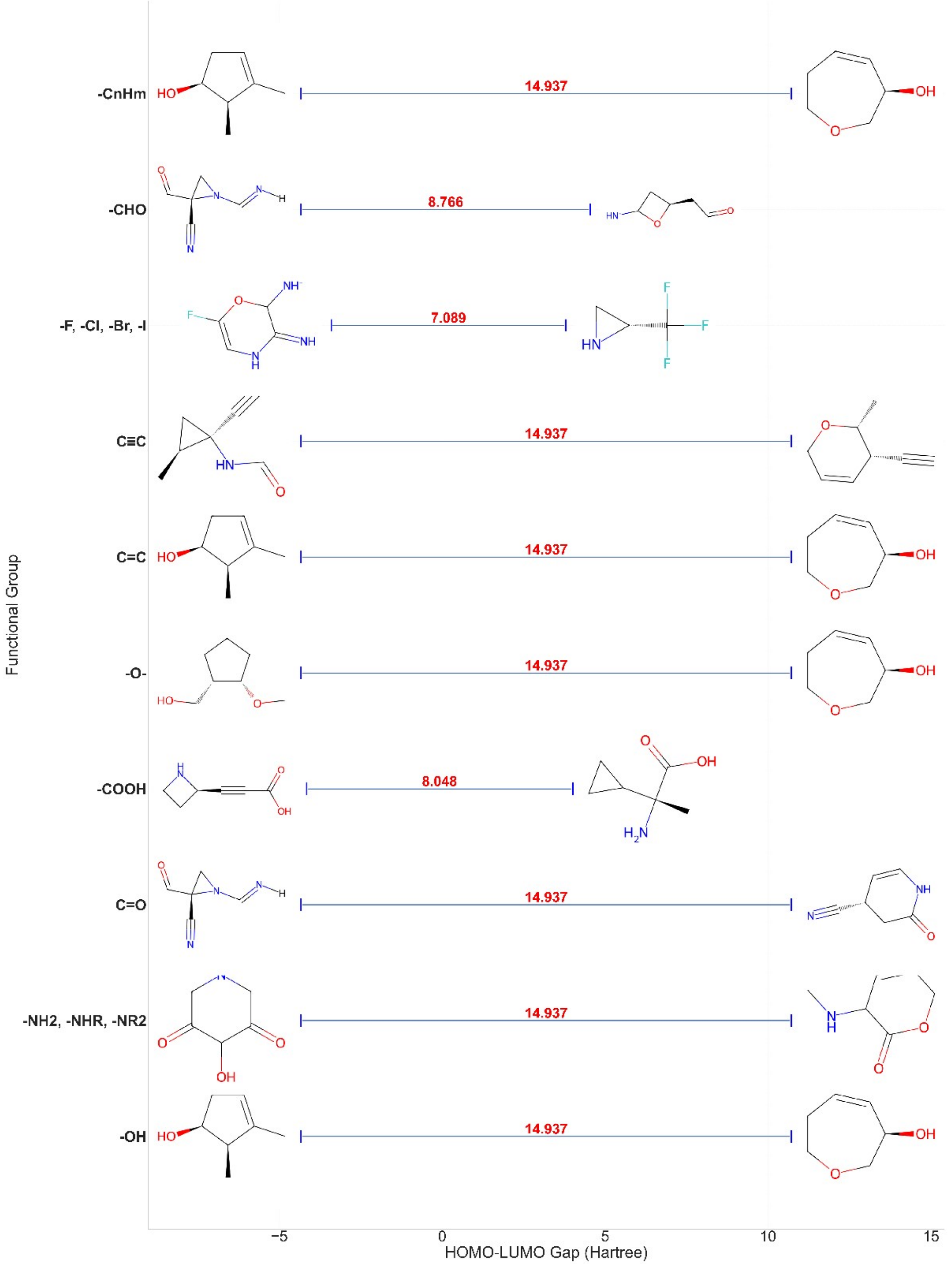

Fig. S16 Visualization of HOMO-LUMO Gaps with Annotated Molecular Structures for Non-PAH (0 Aromatic Rings, 1 Total Rings).

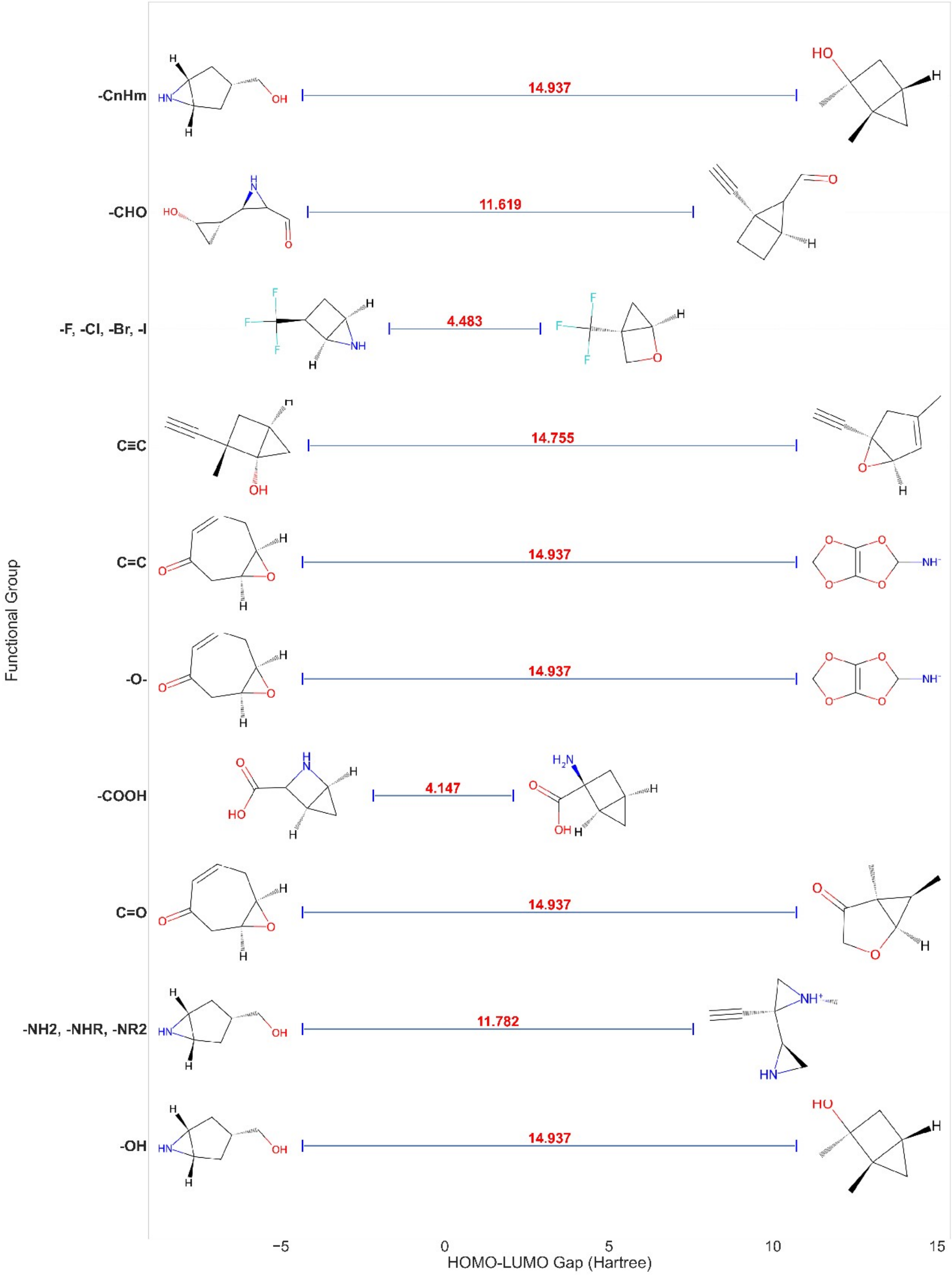

Fig. S17 Visualization of HOMO-LUMO Gaps with Annotated Molecular Structures for Non-PAH (0 Aromatic Rings, 2 Total Rings).

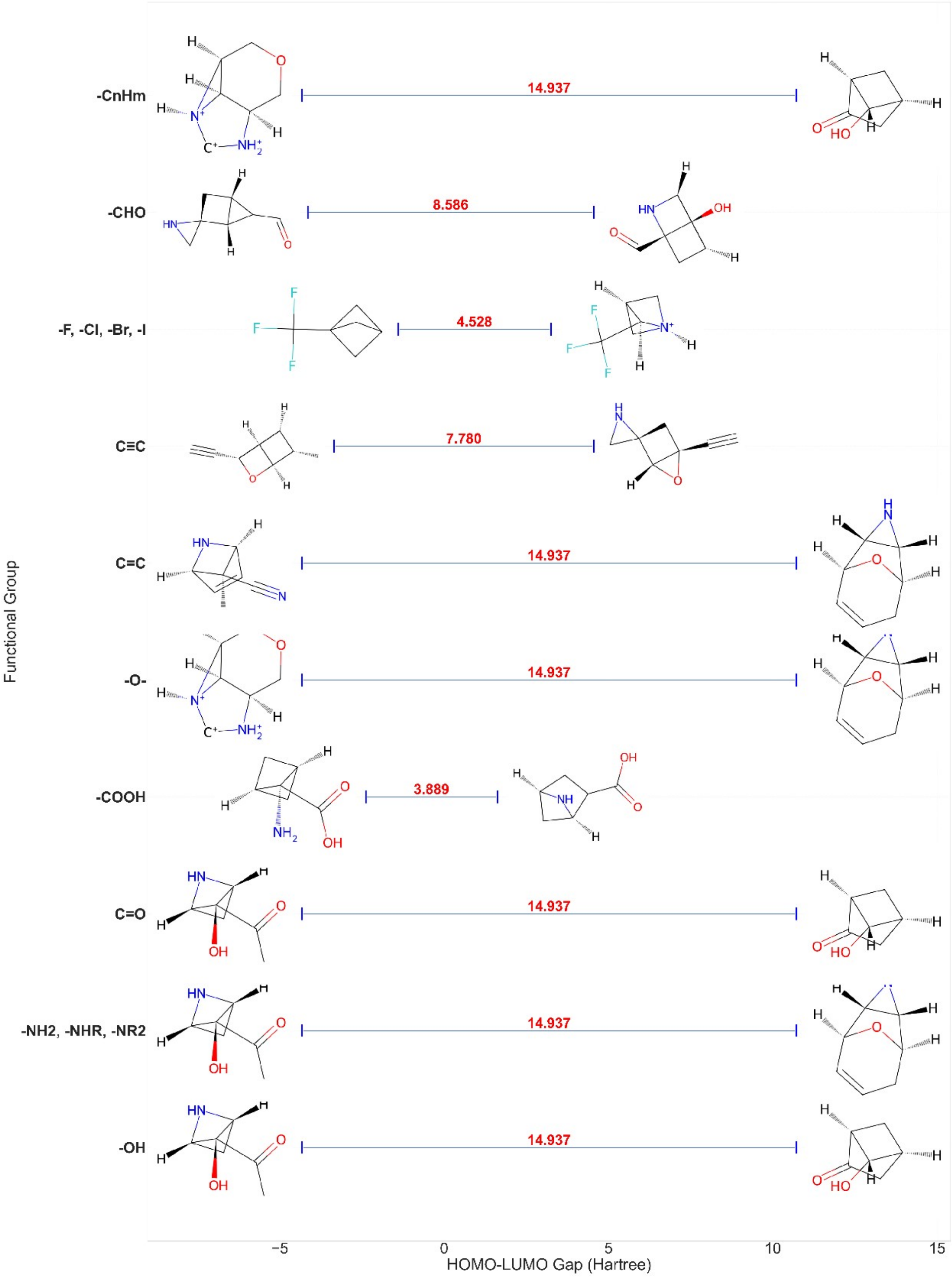

Fig. S18 Visualization of HOMO-LUMO Gaps with Annotated Molecular Structures for Non-PAH (0 Aromatic Rings, 3 Total Rings).

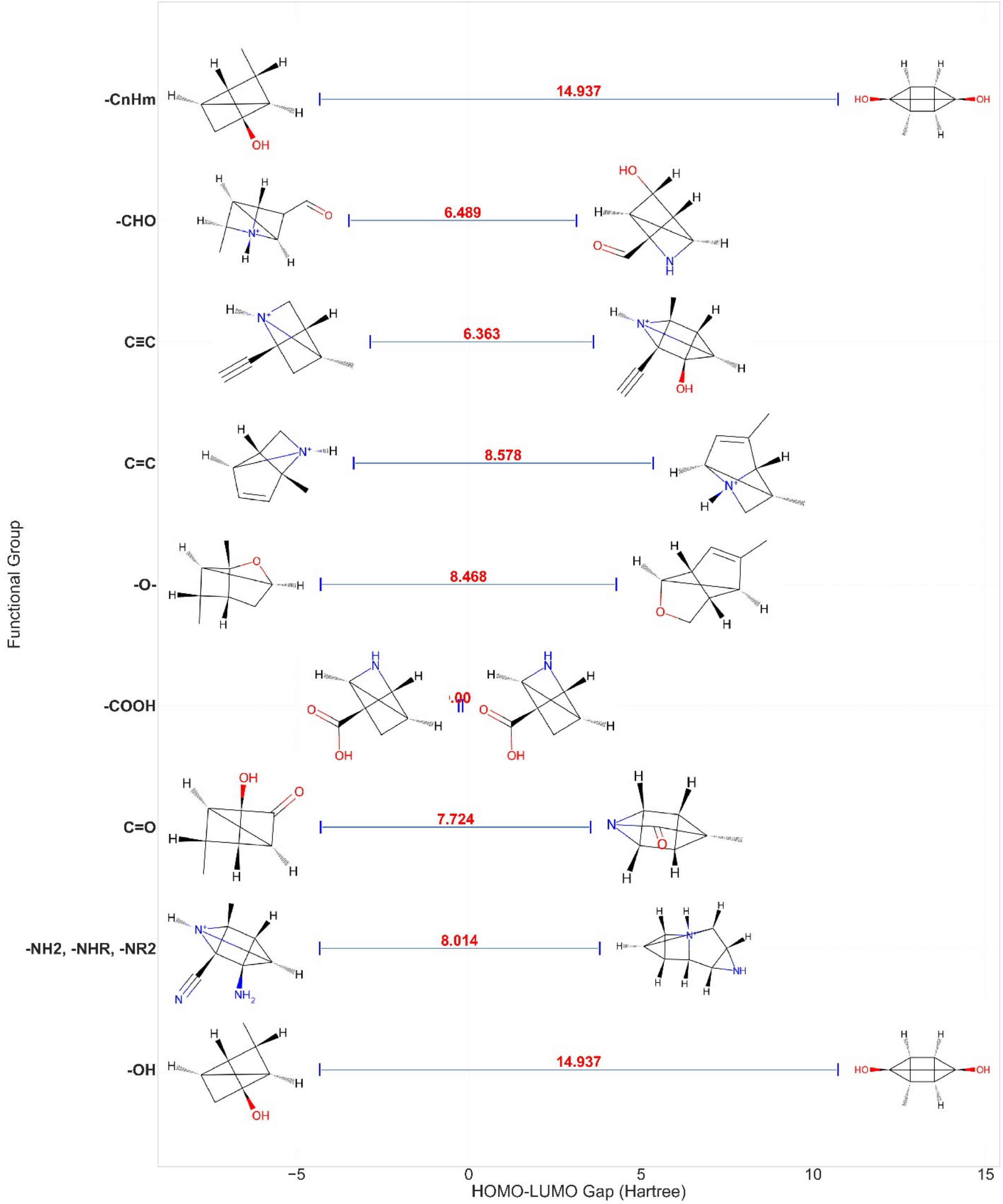

Fig. S19 Visualization of HOMO-LUMO Gaps with Annotated Molecular Structures for Non-PAH (0 Aromatic Rings, 4 Total Rings).

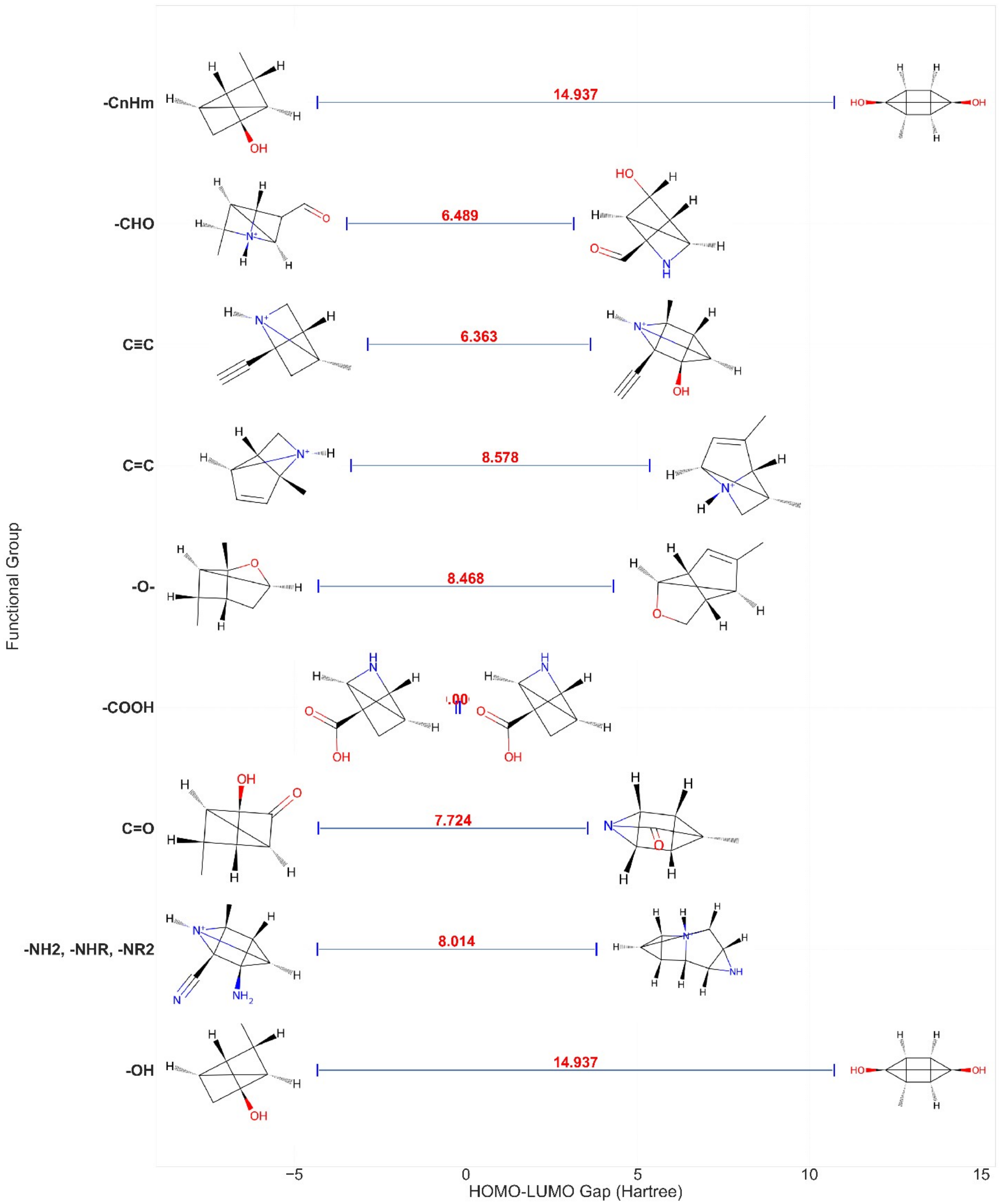

Fig. S20 Visualization of HOMO-LUMO Gaps with Annotated Molecular Structures for Non-PAH (0 Aromatic Rings, 5 Total Rings).

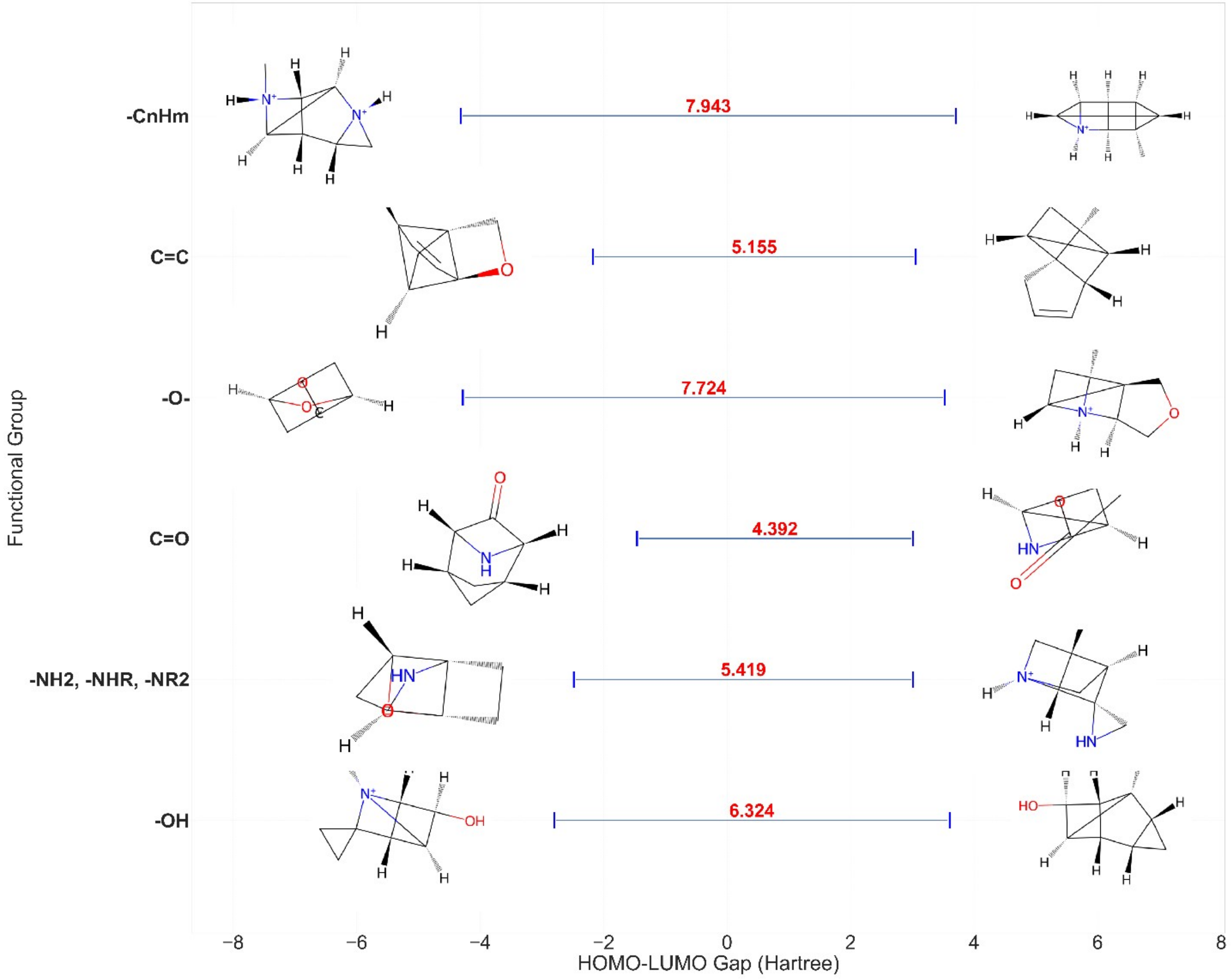

Fig. S21 Visualization of HOMO-LUMO Gaps with Annotated Molecular Structures for Non-PAH (0 Aromatic Rings, 6 Total Rings).

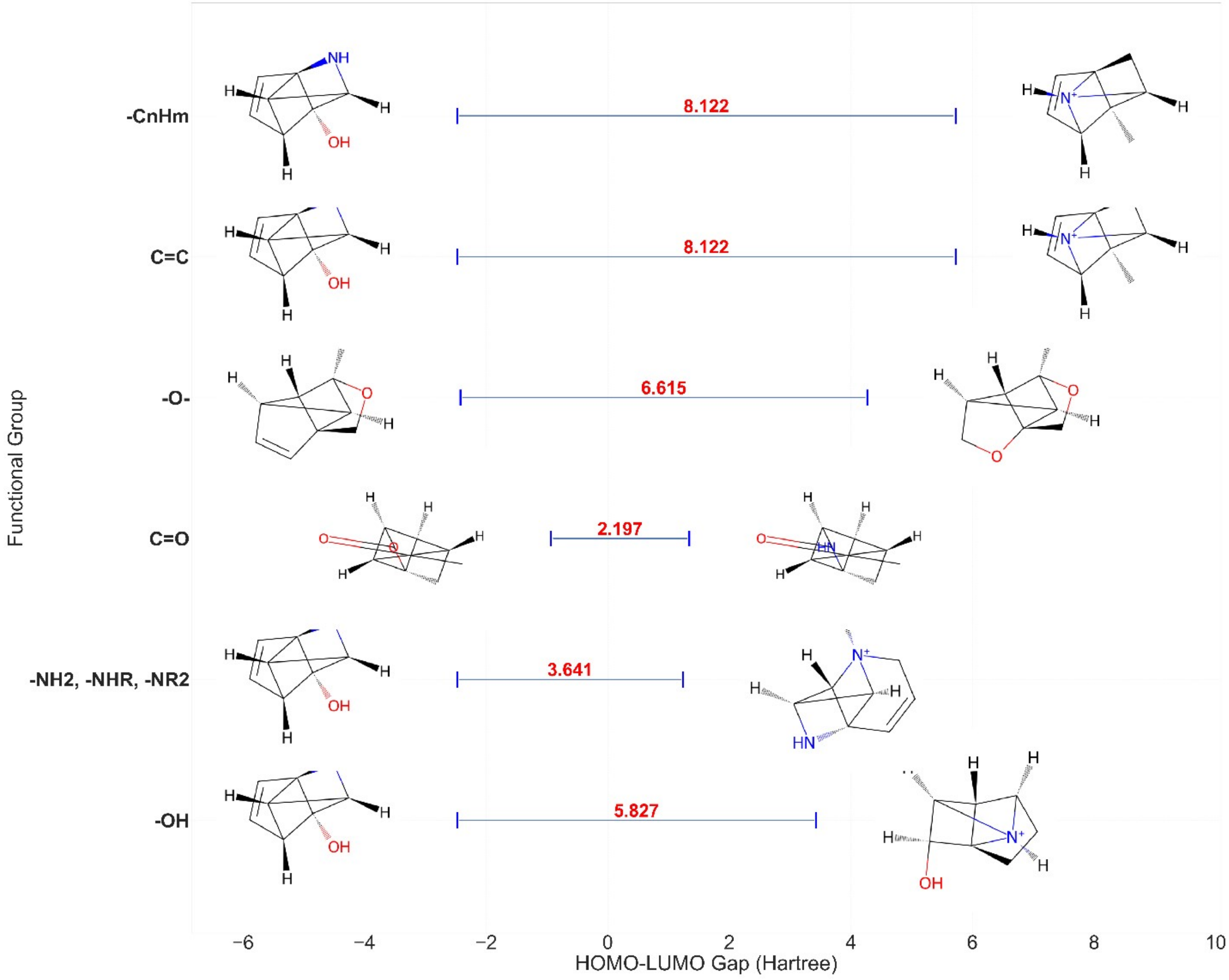

Fig. S22 Visualization of HOMO-LUMO Gaps with Annotated Molecular Structures for Non-PAH (0 Aromatic Rings, 7 Total Rings).

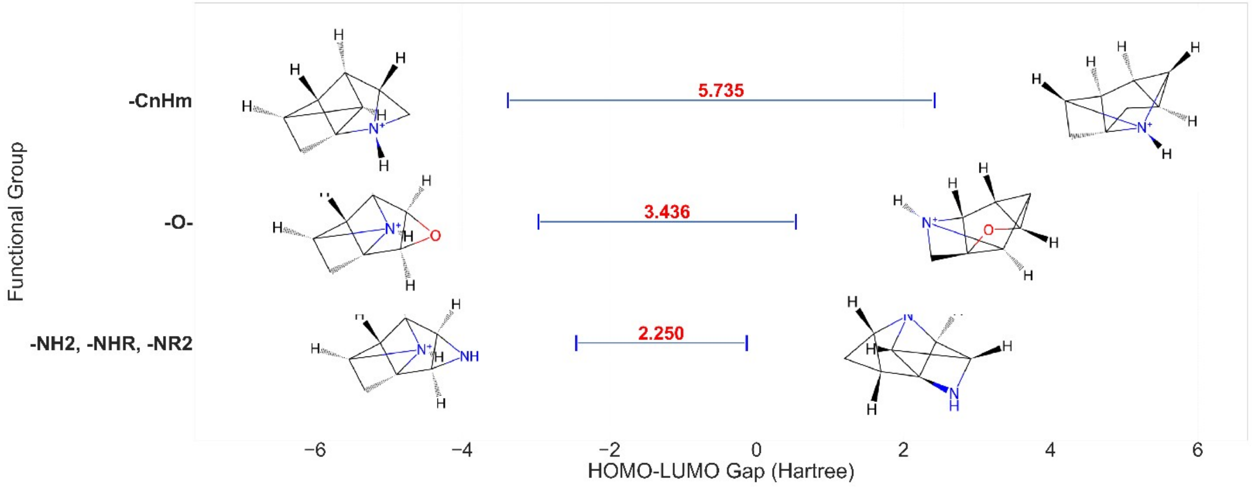

Fig. S23 Visualization of HOMO-LUMO Gaps with Annotated Molecular Structures for Non-PAH (0 Aromatic Rings, 8 Total Rings).

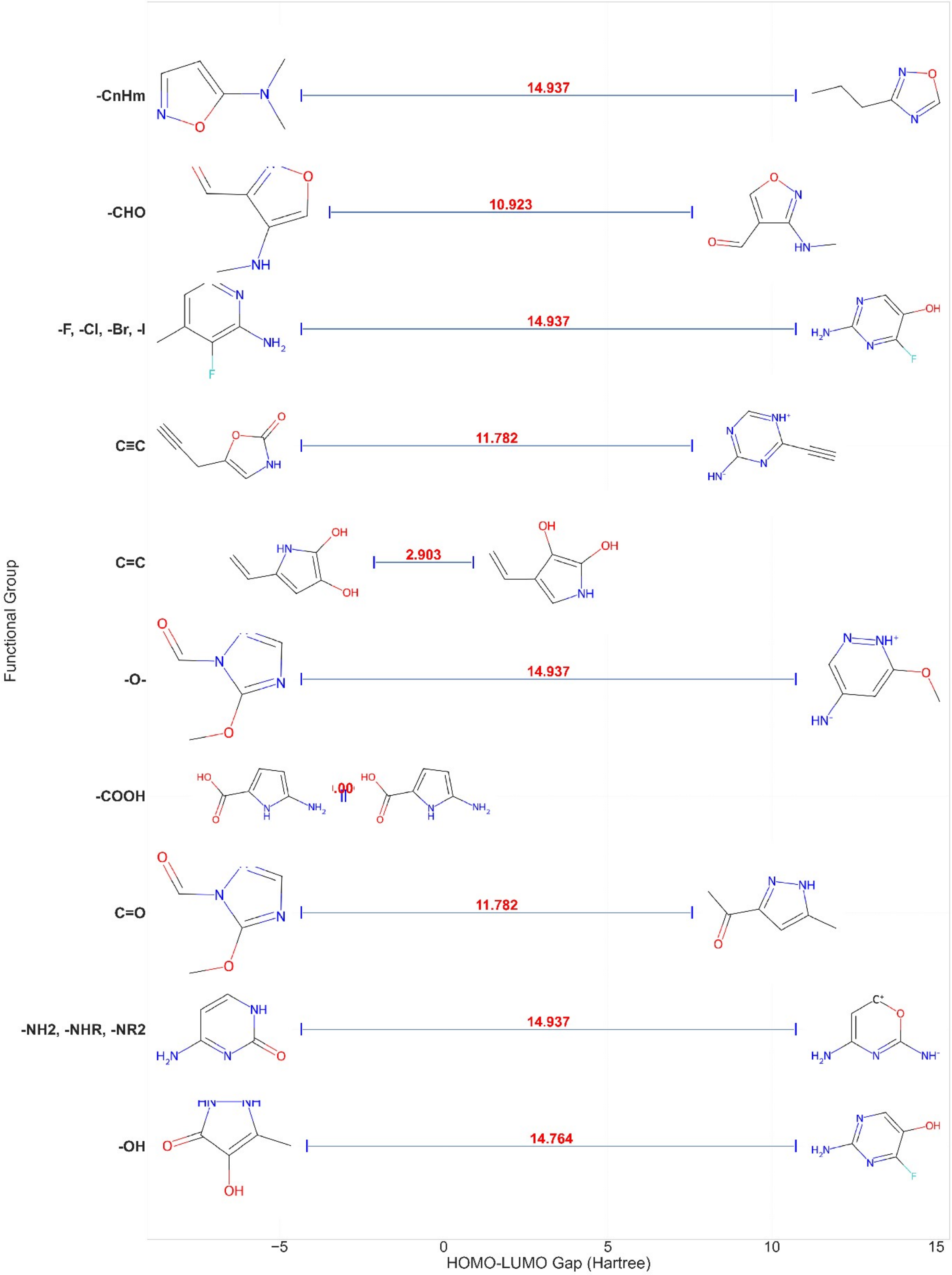

Fig. S24 Visualization of HOMO-LUMO Gaps with Annotated Molecular Structures for Non-PAH (1 Aromatic Rings, 1 Total Rings).

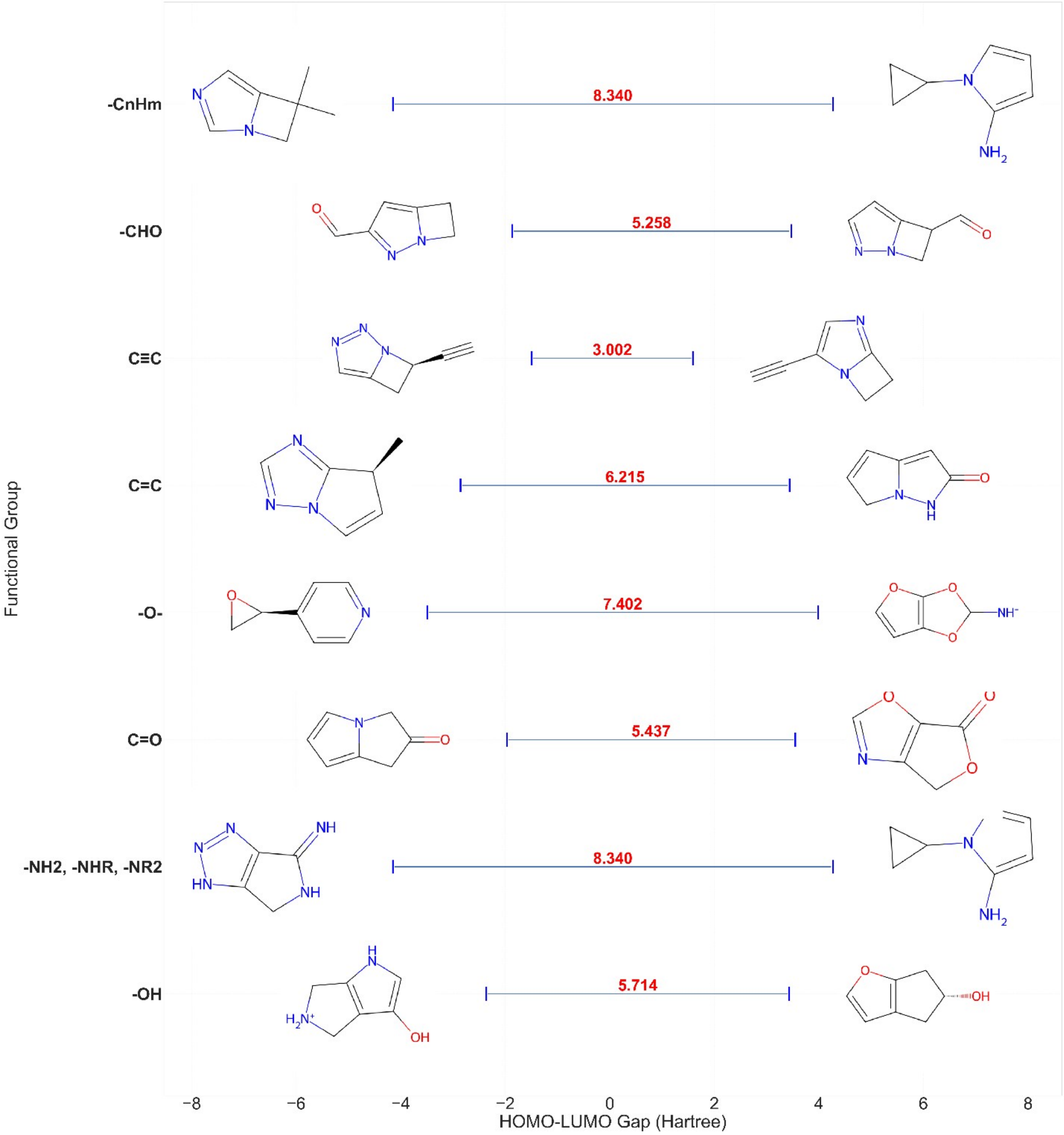

Fig. S25 Visualization of HOMO-LUMO Gaps with Annotated Molecular Structures for Non-PAH (1 Aromatic Rings, 2 Total Rings).

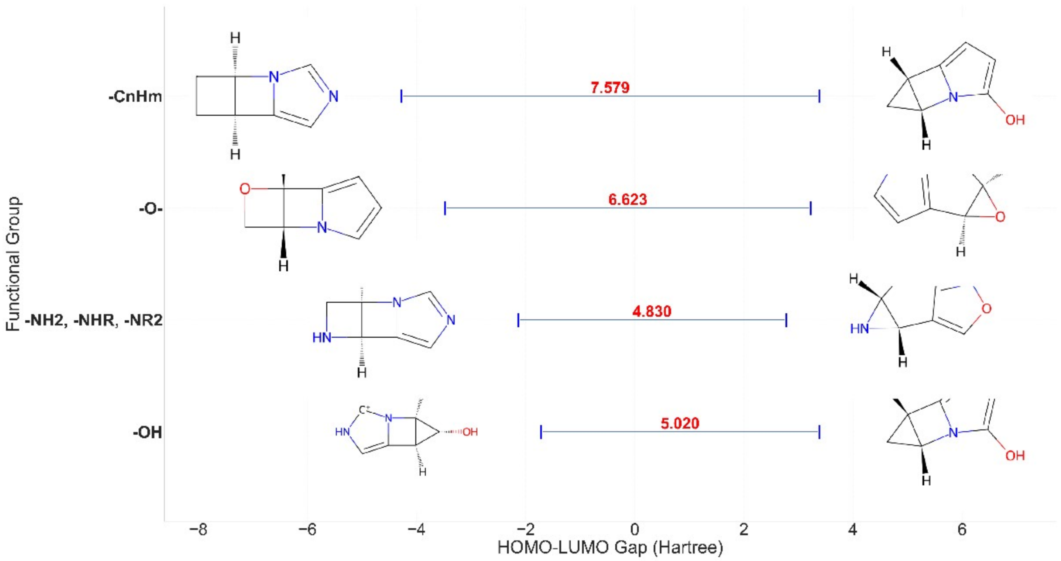

Fig. S26 Visualization of HOMO-LUMO Gaps with Annotated Molecular Structures for Non-PAH (1 Aromatic Rings, 3 Total Rings).

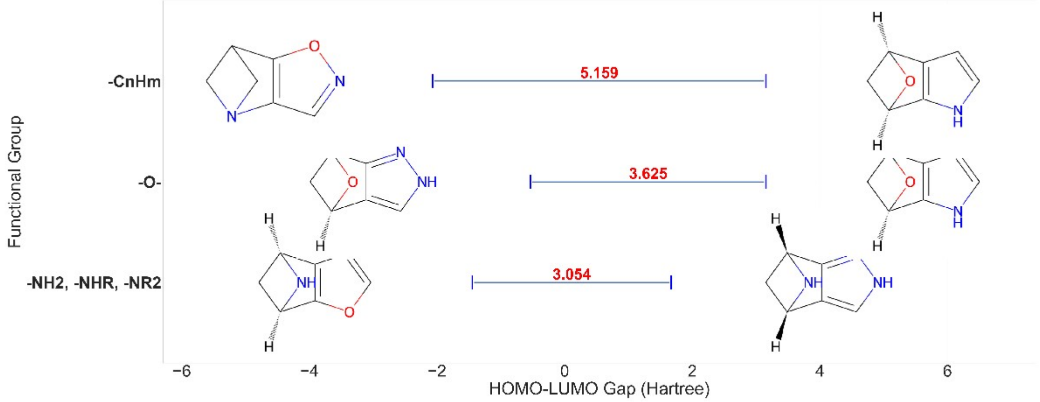

Fig. S27 Visualization of HOMO-LUMO Gaps with Annotated Molecular Structures for Non-PAH (1 Aromatic Rings, 4 Total Rings).

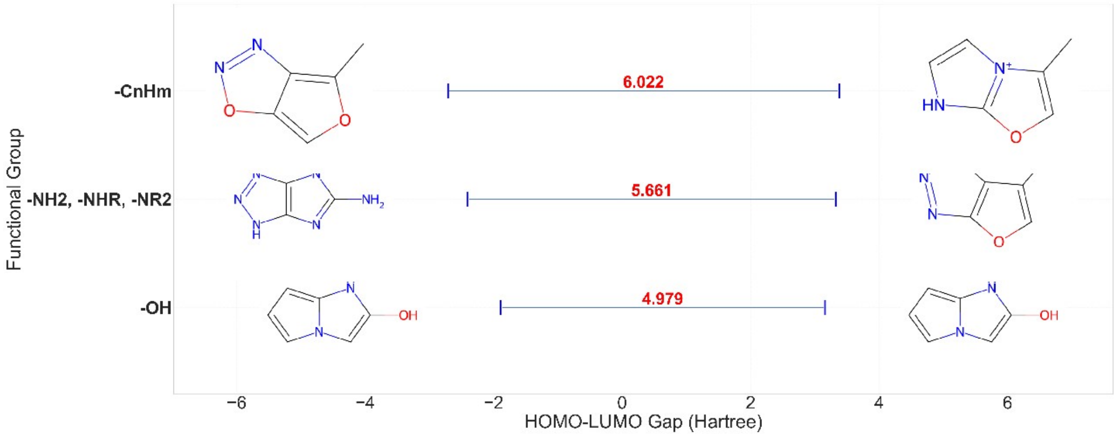

Fig. S28 Visualization of HOMO-LUMO Gaps with Annotated Molecular Structures for Non-PAH (2 Aromatic Rings, 2 Total Rings).

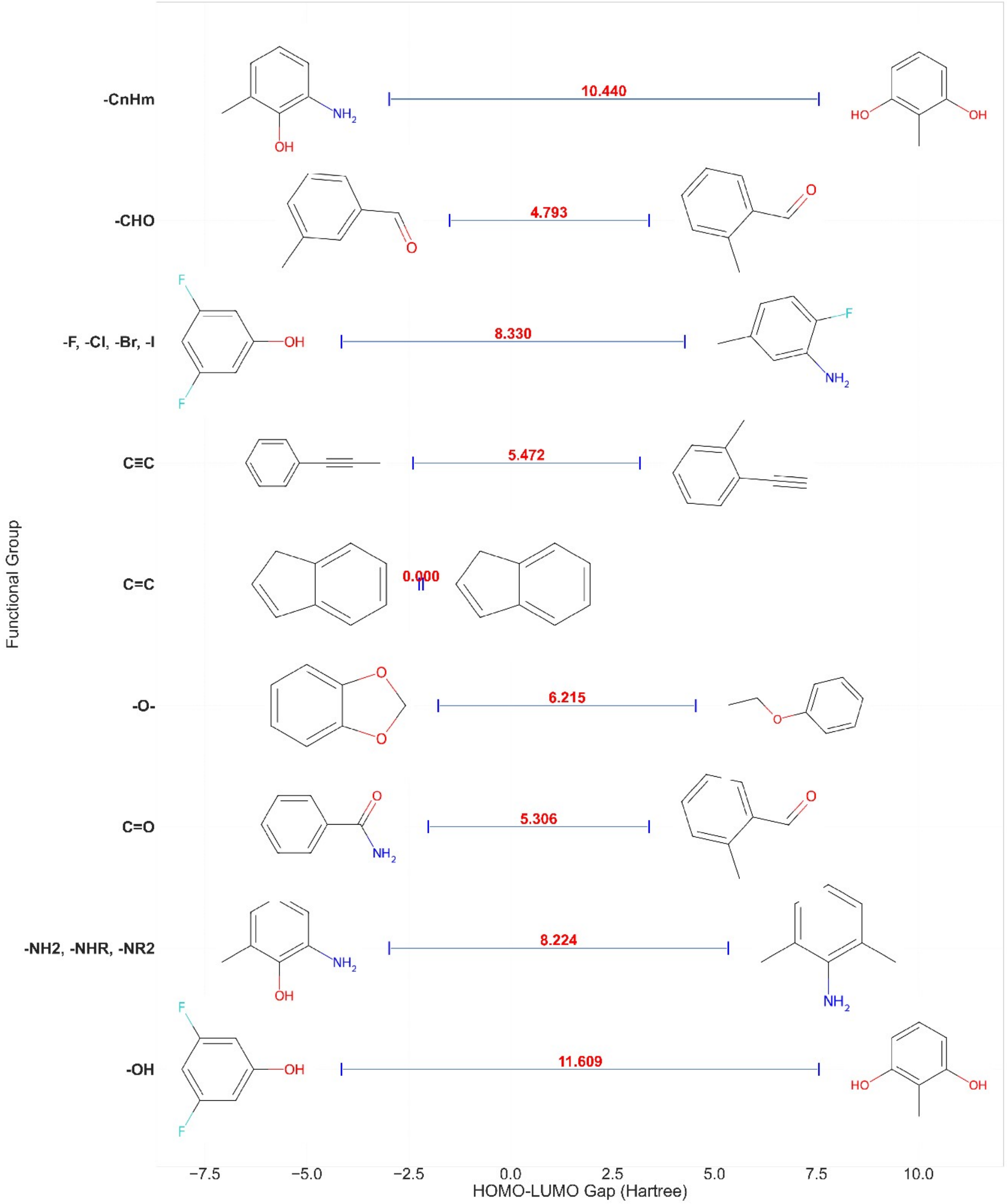

Fig. S29 Visualization of HOMO-LUMO Gaps with Annotated Molecular Structures for PAH-1 (Single Benzene, 1 Aromatic Rings).

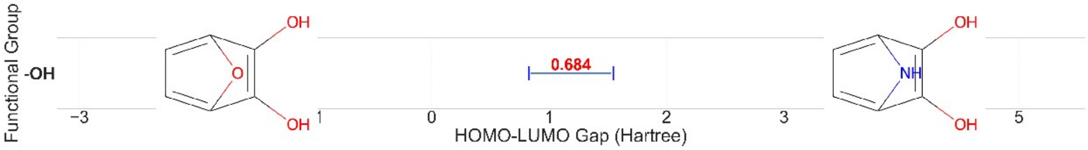

Fig. S30 Visualization of HOMO-LUMO Gaps with Annotated Molecular Structures for PAH-1 (Single Benzene, 2 Aromatic Rings).

Machine Learning Models

In this study, various machine learning regression algorithms were employed to predict HOMO and LUMO energies. These models were selected based on their ability to handle non-linear relationships, manage high-dimensional descriptor spaces, and generalize well across molecular data. The ensemble methods used each contribute unique strengths in terms of robustness, interpretability, and predictive performance. Below is a brief overview of each model:

Adaptive Boosting Regressor (AdaBoostRegressor): AdaBoostRegressor is an ensemble learning method that sequentially trains weak regressors, typically decision trees, and assigns higher weights to previously mis predicted samples. It improves prediction accuracy by adaptively adjusting model parameters to reduce bias and variance.

Bayesian Ridge Regression (BRR): Bayesian Ridge Regression extends linear regression by incorporating Bayesian inference, where the model estimates the probability distribution of regression coefficients using a Gaussian prior. This approach prevents overfitting by regularizing model complexity.

Elastic Net Regression (ENet): Elastic Net combines Lasso (L1) and Ridge (L2) regularization techniques to balance feature selection and coefficient shrinkage. It is particularly effective for datasets with correlated predictors, overcoming the limitations of Lasso's sparsity constraint.

K-Nearest Neighbors Regressor (KNNR): KNNR is a non-parametric algorithm that predicts values based on the average of the k-nearest training samples in the feature space. It is highly flexible but sensitive to noise and outliers.

Linear Regression (LR): Linear Regression is a fundamental statistical method that models the relationship between independent and dependent variables through a linear equation. Despite its simplicity, it serves as a strong baseline model.

Lasso Regression (Least Absolute Shrinkage and Selection Operator, Lasso): Lasso regression applies L1 regularization, which encourages sparsity in regression coefficients by penalizing the absolute values of parameters. This property makes it useful for feature selection.

Multi-Layer Perceptron Regressor (MLPR): MLPR is a neural network-based regressor that learns non-linear relationships through multiple layers of artificial neurons. It uses backpropagation and gradient descent optimization for training and is highly effective for complex datasets.

Ridge Regression (RR): Ridge regression extends linear regression by incorporating L2 regularization, which shrinks regression coefficients toward zero, thereby reducing multicollinearity and overfitting in high-dimensional datasets.

Support Vector Regression (SVR): SVR is a kernel-based regression model that finds an optimal hyperplane in high-dimensional space to minimize error while maintaining generalization. It is robust against outliers and capable of capturing non-linear relationships using kernel functions.

Random Forest (RF): RF is an ensemble learning method that constructs multiple decision trees during training and averages their predictions to reduce variance and prevent overfitting. It is robust against noise and handles high-dimensional data effectively.

Extreme Gradient Boosting (XGBoost, XGB): XGBoost is a gradient-boosting framework that sequentially builds decision trees while minimizing a differentiable loss function. It incorporates regularization techniques to improve generalization and computational efficiency, making it well-suited for structured data.

Extra Trees (ET): Extra Trees builds multiple randomized decision trees by selecting features and split points at random, leading to diverse tree structures. This approach increases variance reduction and enhances generalization compared to standard decision trees.

Gradient Boosting (GB): GB is a boosting technique that iteratively refines weak decision trees by minimizing the residual errors from previous iterations. By optimizing the gradient of the loss function, it effectively reduces bias while maintaining strong predictive power.

Results for 10-Fold Cross-Validation

Table S3 RMSE Results for 10-Fold Cross-Validation: HOMO Energy

Model	Folds										Avg. Train	STD	Test
	1	2	3	4	5	6	7	8	9	10			
AdaBoostReg.	0.884	0.892	0.906	0.888	0.913	0.919	0.895	0.883	0.912	0.898	0.899	0.012	0.900
BayesianRidge	0.910	0.909	0.911	0.912	0.908	0.909	0.909	0.909	0.910	0.910	0.910	0.001	0.910
ElasticNetReg.	1.000	0.999	1.001	1.002	0.999	0.999	0.998	0.999	1.001	1.001	1.000	0.001	1.000
KNeighborsReg.	0.736	0.737	0.739	0.739	0.736	0.736	0.736	0.736	0.739	0.738	0.737	0.001	0.908
LassoReg.	1.000	0.999	1.001	1.002	0.999	0.999	0.998	0.999	1.001	1.001	1.000	0.001	1.000
LinearReg.	0.906	0.906	0.907	0.908	0.905	0.905	0.905	0.905	0.907	0.907	0.906	0.001	0.906
MLPReg.	0.679	0.657	0.674	0.664	0.660	0.672	0.671	0.658	0.682	0.674	0.669	0.008	0.673
RidgeReg.	0.910	0.909	0.911	0.912	0.908	0.909	0.909	0.909	0.910	0.910	0.910	0.001	0.910
SVR	0.920	0.919	0.921	0.922	0.919	0.919	0.919	0.919	0.921	0.920	0.920	0.001	0.920
**HLP-Stack**	**0.000**	**0.000**	**0.000**	**0.000**	**0.000**	**0.000**	**0.000**	**0.000**	**0.000**	**0.000**	**0.000**	**0.000**	**0.000**

Table S4 RMSE Results for 10-Fold Cross-Validation: LUMO Energy

Model	Folds										Avg. Train	STD	Test
	1	2	3	4	5	6	7	8	9	10			
AdaBoostReg.	0.640	0.650	0.644	0.642	0.659	0.645	0.656	0.643	0.652	0.652	0.648	0.006	0.649
BayesianRidge	0.643	0.643	0.642	0.642	0.643	0.642	0.642	0.642	0.643	0.642	0.642	0.000	0.643
ElasticNetReg.	0.943	0.943	0.943	0.943	0.944	0.943	0.943	0.943	0.943	0.943	0.943	0.000	0.943
KNeighborsReg.	0.621	0.621	0.621	0.621	0.621	0.621	0.621	0.621	0.621	0.621	0.621	0.000	0.764
LassoReg.	1.000	0.999	1.000	0.999	1.001	1.000	1.001	0.999	1.000	1.001	1.000	0.001	1.000
LinearReg.	0.594	0.594	0.593	0.594	0.594	0.594	0.594	0.594	0.594	0.593	0.594	0.000	0.594
MLPReg.	0.545	0.544	0.540	0.534	0.539	0.534	0.543	0.523	0.537	0.526	0.537	0.007	0.539
RidgeReg.	0.643	0.643	0.642	0.642	0.643	0.642	0.642	0.642	0.643	0.642	0.642	0.000	0.643
SVR	0.649	0.648	0.648	0.648	0.649	0.648	0.648	0.648	0.649	0.647	0.648	0.000	0.648
**HLP-Stack**	**0.000**	**0.000**	**0.000**	**0.000**	**0.000**	**0.000**	**0.000**	**0.000**	**0.000**	**0.000**	**0.000**	**0.000**	**0.000**

Table S5 MAE Results for 10-Fold Cross-Validation: HOMO Energy

Model	Folds										Avg. Train	STD	Test
	1	2	3	4	5	6	7	8	9	10			
AdaBoostReg.	0.717	0.738	0.719	0.739	0.725	0.747	0.755	0.712	0.717	0.744	0.731	0.015	0.731
BayesianRidge	0.651	0.652	0.651	0.652	0.652	0.650	0.650	0.651	0.650	0.652	0.651	0.001	0.651
ElasticNetReg.	0.708	0.709	0.708	0.709	0.709	0.707	0.707	0.707	0.707	0.709	0.708	0.001	0.708
KNeighborsReg.	0.543	0.544	0.543	0.544	0.544	0.542	0.542	0.543	0.541	0.544	0.543	0.001	0.668
LassoReg.	0.708	0.709	0.708	0.709	0.709	0.707	0.707	0.707	0.707	0.709	0.708	0.001	0.708
LinearReg.	0.648	0.649	0.649	0.649	0.650	0.648	0.648	0.648	0.648	0.649	0.649	0.001	0.649
MLPReg.	0.518	0.509	0.495	0.512	0.499	0.492	0.505	0.507	0.493	0.517	0.505	0.009	0.507
RidgeReg.	0.651	0.652	0.651	0.652	0.652	0.650	0.650	0.651	0.650	0.542	0.651	0.001	0.651
SVR	0.642	0.643	0.642	0.643	0.643	0.641	0.641	0.642	0.641	0.643	0.642	0.001	0.642
**HLP-Stack**	**0.000**	**0.000**	**0.000**	**0.000**	**0.000**	**0.000**	**0.000**	**0.000**	**0.000**	**0.000**	**0.000**	**0.000**	**0.000**

Table S6 MAE Results for 10-Fold Cross-Validation: LUMO Energy

Model	Folds										Avg. Train	STD	Test
	1	2	3	4	5	6	7	8	9	10			
AdaBoostReg.	0.554	0.563	0.554	0.553	0.572	0.557	0.567	0.556	0.566	0.565	0.651	0.007	0.561
BayesianRidge	0.517	0.517	0.516	0.517	0.517	0.517	0.517	0.517	0.517	0.516	0.517	0.000	0.517
ElasticNetReg.	0.774	0.774	0.774	0.774	0.775	0.774	0.775	0.774	0.774	0.774	0.774	0.000	0.774
KNeighborsReg.	0.488	0.438	0.488	0.488	0.488	0.488	0.488	0.488	0.489	0.489	0.488	0.000	0.604
LassoReg.	0.819	0.819	0.819	0.819	0.820	0.819	0.820	0.819	0.820	0.820	0.819	0.000	0.819
LinearReg.	0.472	0.472	0.472	0.472	0.472	0.472	0.473	0.472	0.472	0.472	0.472	0.000	0.472
MLPReg.	0.433	0.433	0.429	0.422	0.429	0.422	0.432	0.414	0.423	0.412	0.425	0.007	0.426
RidgeReg.	0.517	0.517	0.516	0.517	0.517	0.517	0.517	0.517	0.517	0.516	0.517	0.000	0.517
SVR	0.511	0.511	0.511	0.511	0.511	0.511	0.511	0.511	0.511	0.511	0.511	0.000	0.511
**HLP-Stack**	**0.000**	**0.000**	**0.000**	**0.000**	**0.000**	**0.000**	**0.000**	**0.000**	**0.000**	**0.000**	**0.000**	**0.000**	**0.000**

Table S7 R^2 Results for 10-Fold Cross-Validation: HOMO Energy

Model	Folds										Avg. Train	STD	Test
	1	2	3	4	5	6	7	8	9	10			
AdaBoostReg.	0.219	0.203	0.181	0.214	0.165	0.153	0.197	0.217	0.171	0.196	0.192	0.022	0.190
BayesianRidge	0.173	0.172	0.172	0.172	0.173	0.172	0.172	0.172	0.174	0.174	0.173	0.001	0.172
ElasticNetReg.	0.000	0.000	0.000	0.000	0.000	0.000	0.000	0.000	0.000	0.000	0.000	0.000	0.000
KNeighborsReg.	0.458	0.456	0.455	0.456	0.457	0.456	0.457	0.457	0.455	0.456	0.456	0.001	0.175
LassoReg.	0.000	0.000	0.000	0.000	0.000	0.000	0.000	0.000	0.000	0.000	0.000	0.000	0.000
LinearReg.	0.179	0.179	0.179	0.179	0.180	0.178	0.178	0.178	0.180	0.180	0.179	0.001	0.178
MLPReg.	0.539	0.568	0.547	0.560	0.563	0.547	0.548	0.566	0.537	0.547	0.552	0.011	0.547
RidgeReg.	0.173	0.172	0.172	0.172	0.173	0.172	0.172	0.172	0.174	0.174	0.173	0.001	0.172
SVR	0.154	0.154	0.154	0.154	0.155	0.154	0.153	0.153	0.155	0.155	0.154	0.001	0.154
**HLP-Stack**	**1.000**	**1.000**	**1.000**	**1.000**	**1.000**	**1.000**	**1.000**	**1.000**	**1.000**	**1.000**	**1.000**	**1.000**	**1.000**

Table S8 R^2 Results for 10-Fold Cross-Validation: LUMO Energy

Model	Folds										Avg. Train	STD	Test
	1	2	3	4	5	6	7	8	9	10			
AdaBoostReg.	0.590	0.577	0.584	0.588	0.567	0.584	0.571	0.586	0.575	0.576	0.580	0.007	0.579
BayesianRidge	0.587	0.586	0.588	0.587	0.588	0.588	0.588	0.587	0.587	0.589	0.587	0.001	0.587
ElasticNetReg.	0.111	0.110	0.111	0.110	0.111	0.111	0.111	0.110	0.111	0.112	0.111	0.001	0.111
KNeighborsReg.	0.614	0.613	0.614	0.614	0.616	0.615	0.615	0.614	0.614	0.615	0.614	0.001	0.416
LassoReg.	0.000	0.000	0.000	0.000	0.000	0.000	0.000	0.000	0.000	0.000	0.000	0.000	0.000
LinearReg.	0.648	0.647	0.648	0.647	0.648	0.647	0.648	0.647	0.648	0.649	0.648	0.000	0.647
MLPReg.	0.703	0.703	0.708	0.715	0.710	0.715	0.706	0.726	0.712	0.724	0.712	0.008	0.710
RidgeReg.	0.587	0.586	0.588	0.587	0.588	0.588	0.588	0.587	0.587	0.589	0.587	0.001	0.587
SVR	0.580	0.579	0.580	0.580	0.580	0.580	0.580	0.579	0.580	0.581	0.580	0.001	0.580
**HLP-Stack**	**1.000**	**1.000**	**1.000**	**1.000**	**1.000**	**1.000**	**1.000**	**1.000**	**1.000**	**1.000**	**1.000**	**1.000**	**1.000**
